# A Novel Core–Shell Hydrogel 3D Model for Studying Macrophage Mechanosensing and Foreign Body Giant Cell Formation

**DOI:** 10.1002/adhm.202501614

**Published:** 2025-09-20

**Authors:** Manisha Mahanty, Wenquan Ou, Xiaoping Zhu, Jonathan S Bromberg, Xiaoming He, Shaik O. Rahaman

**Affiliations:** ^1^ Department of Nutrition and Food Science University of Maryland College Park MD 20742 USA; ^2^ Fischell Department of Bioengineering University of Maryland College Park MD 20742 USA; ^3^ Department of Veterinary Medicine University of Maryland College Park MD 20742 USA; ^4^ University of Maryland School of Medicine Baltimore MD 21201 USA

**Keywords:** 3D alginate‐collagen microcapsule, biomaterials, foreign body response, giant cell, macrophages

## Abstract

The foreign body response (FBR) to biomaterials is primarily driven by macrophages. At implant sites, macrophages often fuse into destructive foreign body giant cells (FBGCs), yet FBGC‐targeted treatments for FBR remain elusive. To fill this knowledge gap, a novel microscale core–shell hydrogel 3D model is developed using heterogeneous alginate‐collagen microcapsules with varying matrix stiffness to culture macrophages. This 3D model more closely replicates in vivo conditions. This model is further used to investigate the effects of stiffness and TRPV4 (transient receptor potential vanilloid 4) on FBGC formation. Stiffer 3D hydrogel robustly enhances FBGC formation and F‐actin production in wild‐type macrophages compared to softer hydrogel, with IL4 and GMCSF priming amplifying these effects. Crucially, TRPV4‐null macrophages exhibit reduced FBGC formation and F‐actin production, underscoring TRPV4's role in mechanosensing. Further, the N‐terminal residues 1–130 of TRPV4 are identified as critical for FBGC formation and F‐actin generation. RNA‐seq data reveal that TRPV4 modulates inflammatory, fibrotic, and mechanosensitive gene expression in macrophages in 3D environments, offering insights into how TRPV4 governs FBR. Overall, the data establish this 3D model as a powerful tool for biomaterials research and highlight TRPV4 as a key player in macrophage mechanosensing and FBGC formation in 3D condition.

## Graphical abstract

The foreign body response (FBR) to biomaterials is primarily driven by macrophages, which often fuse into destructive foreign body giant cells (FBGCs). To address the limited understanding of FBGC formation, a novel microscale core–shell hydrogel 3D model is developed using heterogeneous alginate‐collagen microcapsules with varying stiffness, offering precise control over physical and biochemical properties and deeper insights into FBR/FBGC formation.

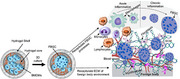

## 1 Introduction

Biomaterials are essential to a wide range of medical applications, including orthopedic and dental implants, as well as cardiovascular and reconstructive surgeries.^[^ ^1^, ^2^, ^3^, ^4^, ^5^, ^6^ ^]^ They function in diverse capacities, serving as prosthetic devices, scaffolds for controlled drug delivery, and matrices to support cell‐based therapies, which represent a new frontier in modern medicine. Despite their extensive use, the FBR to biomaterials remains a significant challenge. This chronic inflammatory reaction occurs when biomaterials (particularly ones that are slowly degradable or non‐degradable) are implanted into soft tissues, often leading to the formation of a fibrous capsule around the implant.^[^ ^2^, ^3^, ^4^, ^5^, ^6^, ^7^, ^8^, ^9^ ^]^ The capsule consists of an inner layer of macrophages and FBGCs and an outer layer of fibrotic tissue. FBR can compromise the functionality and structural integrity of the implant, potentially leading to failure and causing harm or even death in some cases.^[^ ^2^, ^3^, ^4^, ^5^, ^6^, ^7^, ^8^, ^9^ ^]^

Currently, there are no effective therapeutic options to mitigate FBR, which poses a vexing challenge in the field of medicine.^[^ ^2^, ^3^, ^4^, ^5^, ^7^, ^8^, ^9^ ^]^ A deeper understanding of the molecular mechanisms underlying FBR and FBGC formation is crucial for developing new and effective strategies to prevent or reduce these adverse responses. Macrophages are central to the FBR, playing a pivotal role in the inflammatory process.^[^ ^2^, ^5^, ^10^, ^11^, ^12^, ^13^, ^14^, ^15^, ^16^ ^]^ These cells release inflammatory mediators, fuse into multinucleated FBGCs, regulate extracellular matrix (ECM) deposition and turnover, and drive the development of the fibrotic capsule that encases the implant.^[^ ^2^, ^5^, ^10^, ^11^, ^12^, ^13^, ^14^, ^15^, ^16^ ^]^ FBGCs are critical players in the destruction, encapsulation, and/or integration of biomaterials. Therefore, understanding the signaling pathways that regulate macrophage interactions with biomaterials is crucial for advancing the design of biocompatible materials and minimizing the FBR.

Despite significant progress in bioengineering, much of the macrophage research has relied on traditional 2D cultures of macrophages. While valuable, these 2D models do not fully replicate the 3D architecture of tissues, which can influence macrophage behavior, interactions, and functions in ways that differ from in vivo conditions.^[^ ^17^ ^]^ The ability to study immune responses to biomaterials in a 3D context is crucial for understanding the complex interactions that occur in the body and for developing strategies to create more biocompatible materials.

Using heterogeneous alginate‐collagen core–shell hydrogel 3D models offers several advantages in studying FBGC formation. These models provide a more physiologically relevant environment than traditional 2D cultures, allowing for more accurate insights into FBGC formation. The models allow precise control over the microenvironment, including composition, stiffness, and porosity, making it possible to study how different physical and chemical properties affect FBGC behavior. Additionally, the 3D structure of the core–shell hydrogel facilitates better spatial analysis, leading to a more detailed understanding of FBGC morphology and dynamics. These features make in vivo metrics‐generating 3D models a powerful tool for studying the body's response to biomaterials.

Material stiffness has emerged as a critical factor in regulating various cellular behaviors, including immune activation and FBR.^[^ ^10^, ^18^, ^19^, ^20^, ^21^ ^]^ Compliance mismatch between an implant and the surrounding tissue is believed to be a major driver of FBR.^[^ ^10^, ^22^, ^23^ ^]^ At the cellular level, research done in our laboratory and others has shown that macrophages cultured on softer hydrogels exhibit reduced inflammatory activation compared to those on stiffer substrates.^[^ ^10^, ^11^, ^15^, ^24^, ^25^, ^26^ ^]^ This suggests that changes in the stiffness of the implant or surrounding matrix may play a significant role in FBR. Previous studies, including work from our laboratory, have shown that substrate stiffness influences macrophage behavior, regulating their differentiation, fusion into FBGCs, and expression of inflammatory markers.^[^ ^10^, ^11^, ^15^, ^25^, ^26^, ^27^, ^28^, ^29^, ^30^, ^31^ ^]^ Furthermore, subcutaneous implantation of softer hydrogels has been shown to recruit fewer macrophages and result in a less severe FBR, indicating that stiffness can regulate tissue repair responses in vivo.^[^ ^10^, ^24^, ^26^ ^]^

Although much is known about how macrophages become activated and contribute to the FBR, the intracellular signaling events that govern their interactions with implanted materials remain unclear. Defining the receptors that sense biomechanical cues and mapping their downstream pathways is a key unmet need. Our group has identified the ion channel TRPV4 as a critical mediator of implant‐induced responses, including the generation of foreign body giant cells. Our previous research has shown that TRPV4 is essential for FBR and FBGC formation.^[^ ^11^, ^15^ ^]^ Our recent studies demonstrate that TRPV4 is required for both implant stiffness‐driven FBR in vivo and the stiffening of peri‐implant tissue.^[^ ^10^ ^]^ Nevertheless, the mechanisms by which TRPV4 regulates this response remain unresolved. TRPV4, a member of the TRPV superfamily, is broadly expressed across various cell types, including macrophages.^[^ ^10^, ^11^, ^32^, ^33^, ^34^, ^35^, ^36^ ^]^ A broad spectrum of biochemical and physical inputs has been shown to trigger TRPV4 activity, including alterations in extracellular rigidity, fluid balance, applied forces, and soluble factors.^[^ ^10^, ^32^, ^33^, ^34^, ^35^, ^36^, ^37^, ^38^ ^]^ Loss of TRPV4 expression in mouse models leads to multiple defects, encompassing vascular relaxation, regulation of osmotic balance, peripheral nerve function, and tissue remodeling in the lung and skin.^[^ ^39^, ^40^, ^41^, ^42^, ^43^, ^44^ ^]^ Collectively, these findings suggest that TRPV4 plays a crucial role in immune cell activation and the FBR to biomaterials. However, its specific role in regulating FBGC formation within a 3D environment has not yet been explored.

To address this, we developed a heterogeneous core–shell hydrogel 3D in vitro model of alginate and collagen with varying stiffness levels to study the effects of stiffness and the role of TRPV4 in FBGC formation. This 3D model closely replicates the key metrics of in vivo conditions, showing substantial differences from 2D cultures. Our study with this 3D model revealed a significant increase in FBGC formation and F‐actin production in stiffer 3D alginate hydrogels compared to softer ones in wild‐type macrophages. These effects were amplified by priming with IL4 and GMCSF and were less pronounced in TRPV4 null macrophages, emphasizing the role of TRPV4 in stiffness‐sensitive FBGC formation. We also identified that the N‐terminal residues 1–130 of TRPV4 are crucial for FBGC formation and F‐actin generation. Additionally, RNA‐seq data suggested that TRPV4 modulates the expression of inflammatory, fibrotic, and mechanosensitive genes in macrophages within a 3D context, providing insights into how TRPV4 activity regulates FBR. These findings underscore the potential of our 3D model as a tool for biomaterial testing and suggest that TRPV4 plays a critical role in macrophage sensing of extracellular matrix stiffness, driving FBGC formation. This study provides valuable insights for evaluating new biological and synthetic biomaterials for tissue engineering and offers a foundation for developing strategies to mitigate FBR.

## 2 Results

### 2.1 Engineering Heterogeneous Alginate‐Collagen Core–Shell 3D Hydrogel Microcapsules with Varying Core Stiffness

**Figure 1:**
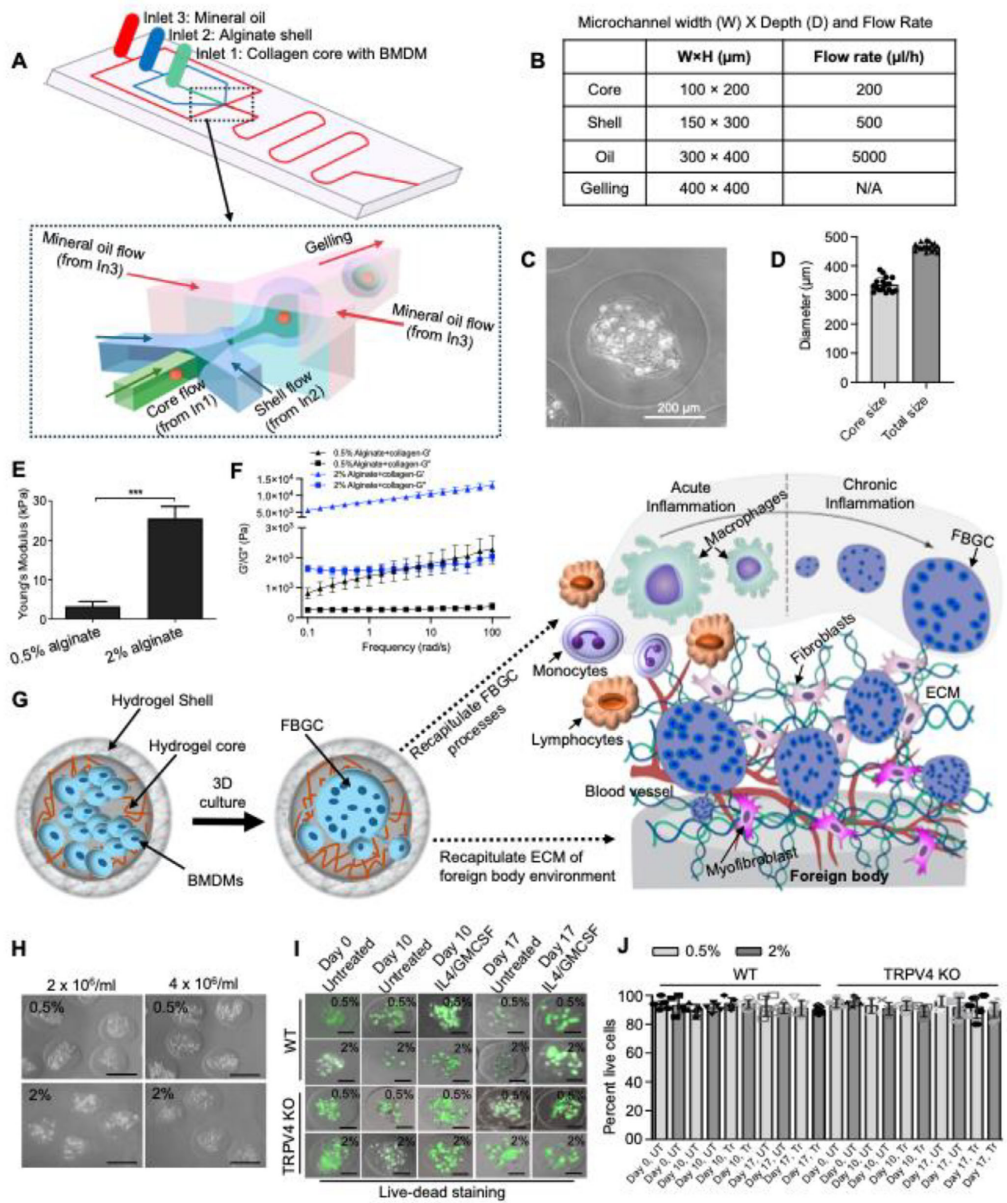
Characterization of heterogenous alginate‐collagen 3D microcapsule cell culture system. A) Image of the nonplanar microfluidic device used for encapsulating BMDMs within 3D microcapsules. B) Table shows the microchannel size and flow rate. C) Representative brightfield image shows encapsulation of BMDMs in 3D hydrogels. D) Core and total size of 3D microcapsules. Core size: 334.5±6.0; total size: 445±4.0; mean ± standard deviation. E) Quantification of the Young's modulus (stiffness) of 3D alginate‐collagen microcapsules with different alginate concentrations (0.5% and 2%), as determined by AFM analysis; n = 50 force curves per sample. Student's t‐test, ***p ≤ 0.001. F) Rheological characterization of the core material in 0.5% and 2% 3D alginate‐collagen microcapsules. Storage modulus G' (triangles) and loss modulus G" (squares) of the core material gels with different alginate percentages (0.5% and 2%) via the frequency sweep test at 1% strain. G) Simplified schematic diagram shows the 3D alginate‐collagen hydrogels and progression of FBR in response to implantation. H) Representative phase contrast images of BMDMs encapsulated in 3D microcapsules with varying alginate concentrations (0.5% and 2%), with cell densities of 2×10⁶ BMDMs/mL and 4×10⁶ BMDMs/mL. Scale bar: 400 µm. I) Representative confocal microscopy images show the viability of encapsulated WT and TRPV4 KO BMDMs, stained with Calcein AM (green = live cells; red = dead cells) on days 0, 10, and 17 within 3D alginate‐collagen microcapsules (0.5% and 2%) cultured with or without IL4 and GMCSF (25 ng mL^−1^) treatment. Scale: 200 µm. J) Graph shows the percentage of live cells from experiment D. Data are presented as the mean of three biological replicates, with five images per group. Statistical analysis was performed using one‐way ANOVA. UT: untreated, and Tr: IL4 plus GMCSF treated.

To create in vivo‐mimicking environment, we leveraged microfluidic technology to engineer heterogeneous 3D hydrogel microcapsules composed of type I collagen and alginate.^[^ ^54^, ^55^ ^]^ The microfluidic device (**Figure** **1**A,B) features three inlets, one for mineral oil, one for alginate, and one for collagen and alginate mixture used to suspend mouse BMDMs. The parameters of microchannels within the microfluidic device and their corresponding flow rates during 3D hydrogel formation are listed in Figure 1B. The outer shell of the microcapsule is made of pure alginate hydrogel, while the core is a hybrid gel of both alginate and collagen embedded with the BMDMs (Figure 1C). After encapsulation, the 3D hydrogels exhibit a distinct core–shell structure, showing the successful encapsulation of BMDMs in the core (Figure 1C). Within the 3D hydrogels, the core has a size of 335.1 ± 24.3 µm, while the total size of the 3D hydrogels measures 466.1 ± 13.7 µm (Figure 1D). To utilize this 3D microcapsule model for studying stiffness‐dependent FBGC formation in vitro, we produced microcapsules with varying stiffness levels, mimicking that of normal tissue (≈1 kPa) and fibrotic tissue (≈8–50 kPa) by adjusting the alginate concentration in the core while maintaining the same collagen concentration.^[^ ^10^, ^27^, ^28^ ^]^ The stiffness of the microcapsules was confirmed via atomic force microscopy (AFM) analysis, with AFM force curves demonstrating that 3D microcapsules with 2% (stiff) alginate were ten times stiffer than those with 0.5% (soft) alginate (Figure 1E; Figure S1, Supporting Information). The stiffness of both soft and stiff microcapsules was measured at days 0 and 17 using atomic force microscopy. No significant change in stiffness was observed over time in either group (P = 0.22) (Figure S2, Supporting Information). The dynamic mechanical properties of core materials were further measured using a rheometer. As shown in Figure 1F, the storage modulus (G′) was significantly higher than the loss modulus (G″) across the frequency range of 0.01–100 Hz, indicating the formation of a stable 3D network. As the alginate increased to 2%, both the G′ and G″ values of the core material gels also increased, suggesting the development of stiffer gel structures. Together, these results indicate the successful fabrication of 3D microcapsules with an alginate‐collagen core of adjustable matrix stiffness. A schematic illustration showing that the heterogeneous 3D hydrogel microcapsules replicate a 3D architecture of a fibrotic tissue that induces FBGC formation is given in Figure 1G.

To assess the suitability of these engineered 3D microcapsules for viable cell culture and their potential as an in vitro model, we encapsulated BMDMs (2 × 10^6^ and 4 × 10^6^ mL^−1^) within both soft and stiff 3D alginate‐collagen microcapsules. These microcapsules were cultured in complete DMEM, with or without an IL4 and GMCSF (25 ng mL^−1^) fusogenic cocktail, and live‐dead staining was performed on days 0, 10, and 17 (Figure 1H–J). We found that both soft and stiff microcapsules supported the survival of BMDMs. Collectively, these results demonstrate the successful development of a heterogeneous alginate‐collagen core–shell 3D hydrogel microcapsule cell culture system to replicate in vivo conditions of FBGC formation.

### 2.2 3D Alginate‐Collagen Microcapsule Model with a Stiff Core can Closely Replicate the Metrics of FBGC Formation In Vivo

**Figure 2:**
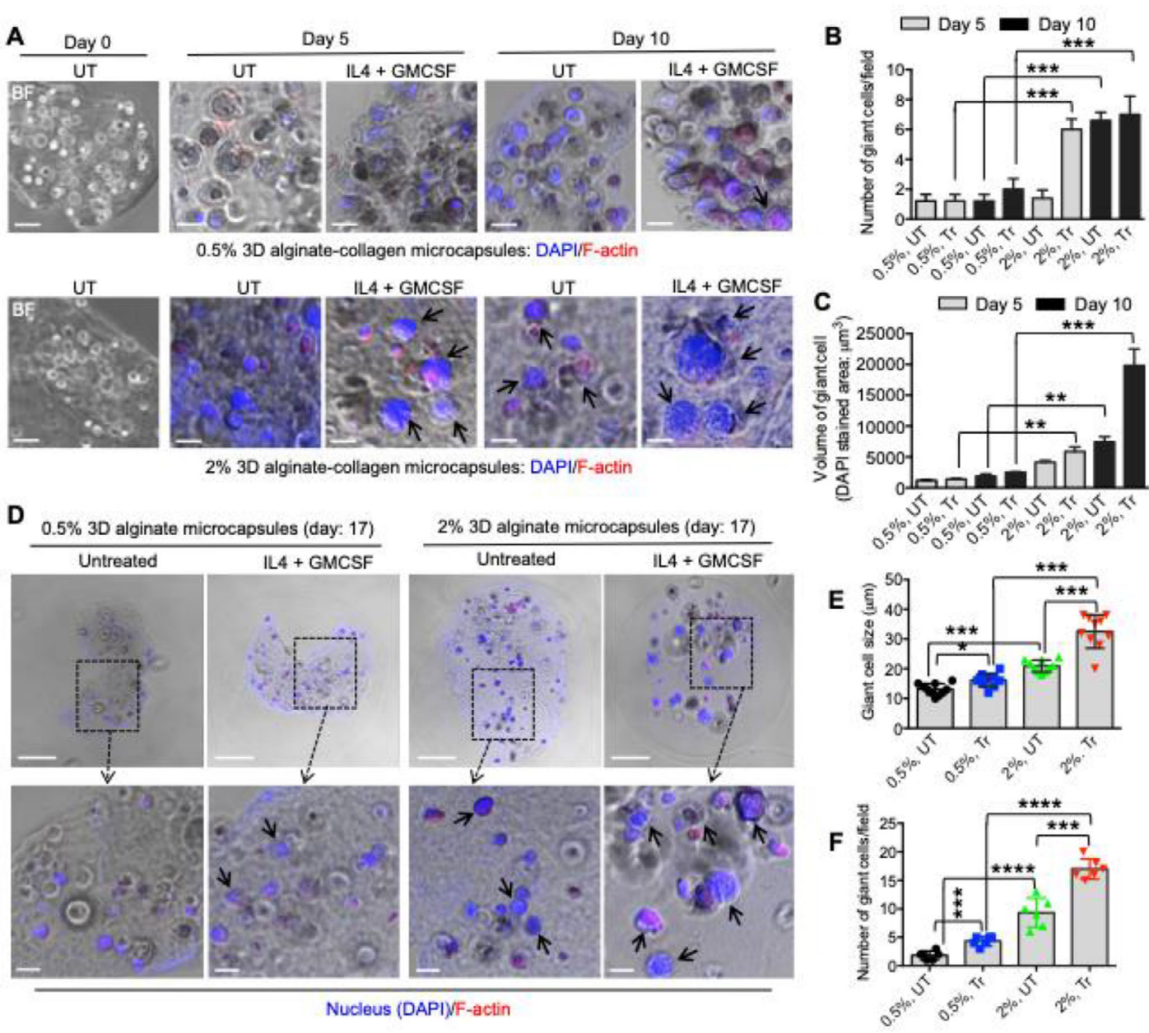
FBGC formation is regulated by both biochemical and matrix stiffness in 3D microcapsules. A) Representative merged phase and fluorescent confocal images of WT BMDMs encapsulated in 3D microcapsules with varying alginate concentrations (0.5% and 2%), stained for F‐actin (red) and nuclei (blue), and treated with (Tr) or without (UT) IL4 and GMCSF (25 ng mL^−1^) on days 0, 5, and 10. B) Quantification of the number of FBGCs per field and C) the volume of FBGCs (µm^3^) are shown for the experiment described in (A). Scale bar: 50 µm. Data are presented as the mean from three biological replicates, with five images analyzed per group; statistical analysis was performed using one‐way ANOVA followed by Bonferroni's test. **p ≤ 0.01, and ***p ≤ 0.001. Maintenance of FBGCs in 3D microcapsules over an extended period. D) Representative immunofluorescent images showing F‐actin (red) and nuclei (blue) of WT BMDMs encapsulated in soft and stiff 3D alginate‐collagen microcapsules, cultured with or without IL4 and GMCSF (25 ng mL^−1^) for 17 days. E) Quantification of FBGC size (µm) and F) the number of giant cells per field from the experiment in (D) are presented. Scale bar: 50 µm. Data are shown as the mean from three biological replicates, with five images analyzed per group; statistical analysis was conducted using one‐way ANOVA followed by Bonferroni's test. *p ≤ 0.05, ***p ≤ 0.001, and ****p ≤ 0.0001.

Both mechanical and biochemical stimuli can significantly influence the severity of FBR and the formation of FBGCs in vivo.^[^ ^10^, ^11^, ^26^ ^]^ To determine whether the 3D alginate‐collagen microcapsule model replicates these in vivo findings, we used both soft and stiff microcapsules with WT BMDMs (4 × 10^6^ mL^−1^) to assess FBGC formation over time. Our results revealed a steady and significant increase in both the number and volume of giant cells in stiff microcapsules compared to soft ones over time (**Figure** **2**A–C). The addition of fusogenic cytokines (IL4 and GMCSF, 25 ng mL^−1^) further amplified the size and volume of giant cells within stiff microcapsules by day 10 (Figure 2A–C). By day 17, treatment with IL4 and GMCSF resulted in a threefold increase in the size of giant cells and a fourfold increase in their number in stiff microcapsules compared to soft ones (Figure 2D–F). These findings from our 3D model are consistent with previous in vivo studies,^[^ ^10^ ^]^ reinforcing the validity of our 3D model for studying FBGC formation.

**Figure 3:**
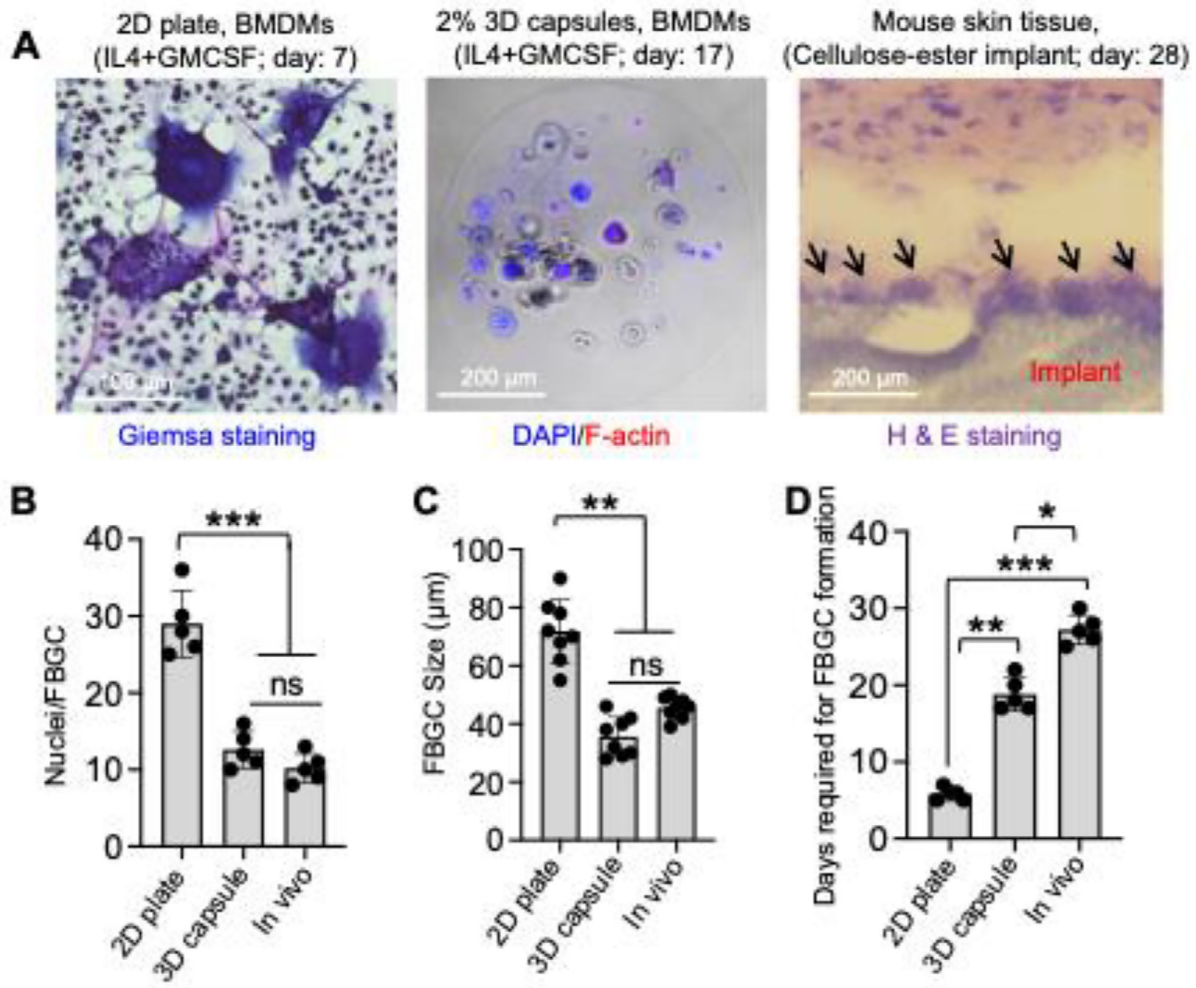
3D model with a stiff core can closely mimic the metrics of FBGC formation in vivo. A) Representative images show FBGC formation in a 2D (left), 3D (middle), and in vivo (right) models. B–D) Bar graphs show properties (nuclei per FBGC, FBGC size, and days required for FBGC formation) of FBGCs generated in 2D, 3D, and in vivo models. Data are presented as the mean from three biological replicates, with five images analyzed per group; statistical analysis was performed using one‐way ANOVA followed by Bonferroni's test. **p ≤ 0.01, ***p ≤ 0.001, and non‐significant is indicated as “ns”.

Using this 3D core–shell hydrogel model, we observed that the nuclei per FBGC (26 in 2D vs 12 in 3D vs 10 in vivo), FBGC size (70 µm in 2D vs 40 µm in 3D vs 50 µm in vivo), and the time required for FBGC formation (6 days in 2D vs 17 days in 3D vs 28 days in the in vivo dermal cellulose‐ester implant model) more closely aligned with in vivo outcomes, demonstrating notable differences from traditional 2D and 3D cultures (**Figure** **3**A–D). For nuclei per FBGC, FBGC size, and the number of days required for FBGC formation, both 3D and in vivo results differed significantly from the 2D system. There were no statistically significant differences between the 3D and in vivo systems for nuclei per FBGC and FBGC size. However, the time required for FBGC formation was significantly longer in the in vivo system compared to the 3D system (Figure 3D). These findings highlight the utility of the 3D core–shell hydrogel model in replicating in vivo macrophage behavior and FBGC formation more accurately than conventional 2D and 3D culture systems. Our 3D model's ability to better mimic the mechanical and biochemical cues of the in vivo environment makes it a valuable tool for studying macrophage responses and the foreign body reaction, offering improved predictive value for preclinical research.

### 2.3 TRPV4‐Mediated Mechanosensation Facilitates Actin Polymerization and FBGC Formation in 3D Alginate‐Collagen Microcapsules

**Figure 4:**
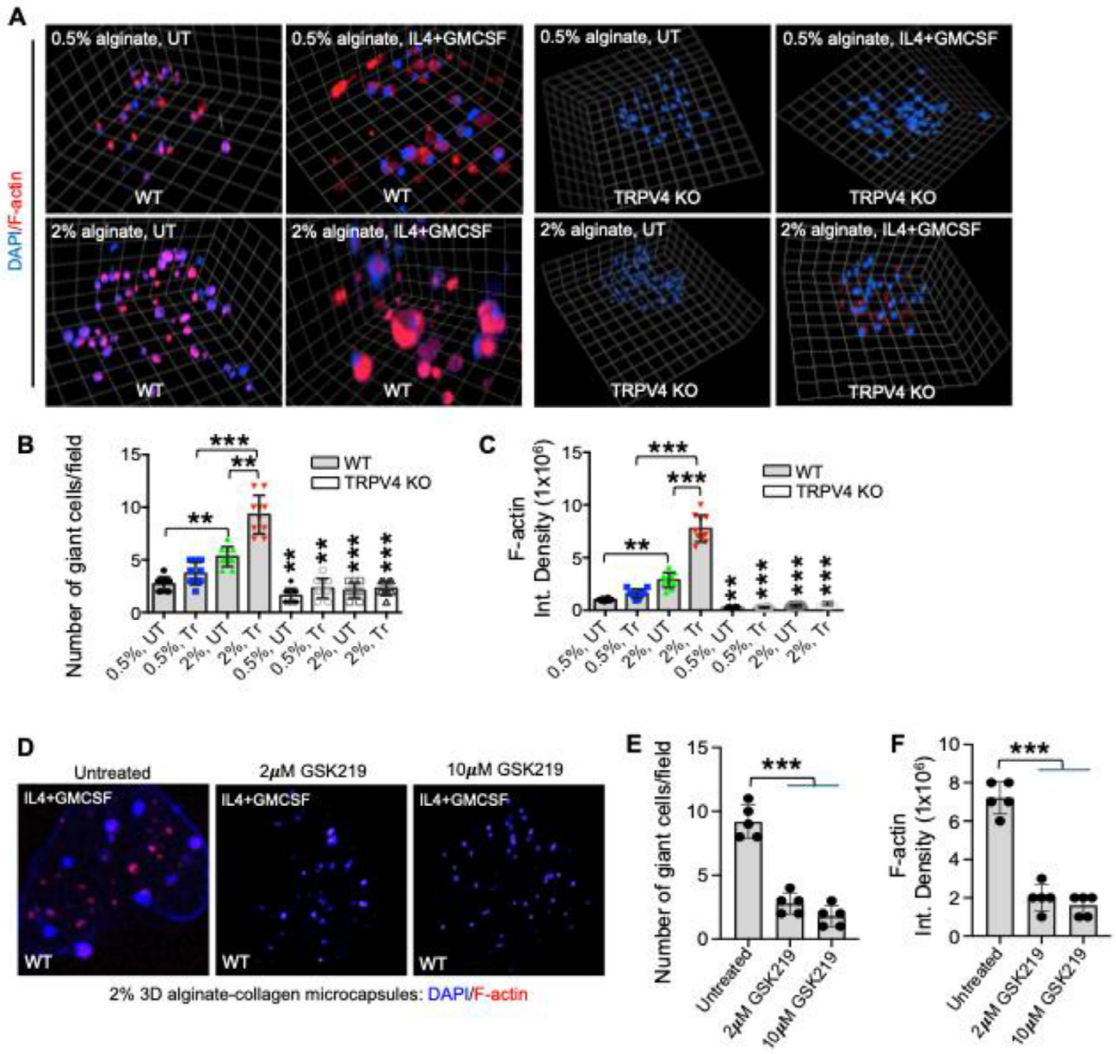
TRPV4 is a key regulator of cytoskeletal remodeling and FBGC formation in response to mechanical and biochemical stimuli in a 3D microcapsule model. A) Representative spinning‐disk confocal microscopy images of WT and TRPV4 KO BMDMs encapsulated in 3D alginate‐collagen microcapsules with different alginate concentrations (0.5% and 2%) after 17 days of culture, with or without IL4 and GMCSF (25 ng mL^−1^). Microcapsules were stained for nuclei (blue) using DAPI and for F‐actin (red) using phalloidin. B) Quantification of the number of giant cells per field. C) Quantification of F‐actin generation as indicated by phalloidin staining from the experiment in (A). Data in figures B and C are presented as the mean from three biological replicates, with ten images analyzed per group; statistical analysis was performed using one‐way ANOVA. **p ≤ 0.01, and ***p ≤ 0.001. Statistical significance in the graphic for TRPV4 KO cells was determined relative to the corresponding condition in WT cells. In Figure 4B,C, the comparisons represented by the latter four bars reflect results from TRPV4 KO cells relative to their corresponding conditions in WT cells. D) Representative spinning‐disk confocal images of multinucleated FBGCs and F‐actin formation in WT BMDMs encapsulated in stiff 3D alginate‐collagen microcapsules following with or without treatment of GSK2193874 (GSK219) (2 and 10 µm). Nuclei are stained with DAPI (blue), and F‐actin is stained with phalloidin (red). E) Quantification of the number of giant cells per field and F) quantification of F‐actin levels from the experiment in (D). Data are presented as the mean from three biological replicates, with 4–7 images analyzed per group; statistical analysis was performed using one‐way ANOVA. ***p ≤ 0.001.

Previous work by our lab and others has demonstrated that TRPV4 regulates intracellular matrix stiffening by driving F‐actin formation and cytoskeletal remodeling, which promotes cell fusion.^[^ ^10^, ^15^, ^56^ ^]^ To explore whether TRPV4 is involved in stiffness‐dependent F‐actin generation in a 3D microcapsule model, we encapsulated WT and TRPV4 KO BMDMs in both soft and stiff microcapsules. After 17 days, we quantified FBGC formation and F‐actin generation using spinning disk confocal microscopy. In WT BMDMs, stiff microcapsules showed a threefold increase in FBGC number per field and a fourfold increase in F‐actin generation compared to soft microcapsules (**Figure** **4**A–C). Conversely, TRPV4 KO BMDMs exhibited reduced FBGC formation and F‐actin generation across all conditions, indicating that TRPV4 is essential for these processes in response to stiffness. Moreover, priming the microcapsules with IL4 and GMCSF for 17 days led to a fivefold increase in FBGC number per field and a sevenfold increase in F‐actin generation in stiff WT microcapsules compared to stiff TRPV4 KO microcapsules (Figure 4A–C). These findings confirm that TRPV4 plays a crucial role in cytoskeletal remodeling and FBGC generation in response to both mechanical and biochemical stimuli in the 3D microcapsule model, further validating this model for studying FBGC formation. To determine whether pharmacological inhibition of TRPV4 using a specific antagonist would suppress FBGC formation, we analyzed FBGC formation by quantifying spinning‐disk confocal images of multinucleated FBGCs and F‐actin formation in WT BMDMs encapsulated in stiff 2% 3D alginate‐collagen microcapsules following with or without treatment of GSK2193874 (2 and 10 µm), a selective TRPV4 antagonist. GSK2193874 treatment exhibited reduced FBGC formation and F‐actin generation across all conditions, indicating that TRPV4 is essential for these processes (Figure 4D–F).

### 2.4 FBGC Formation in 3D Microcapsules Requires N‐Terminal Residues 1–130 of TRPV4

**Figure 5:**
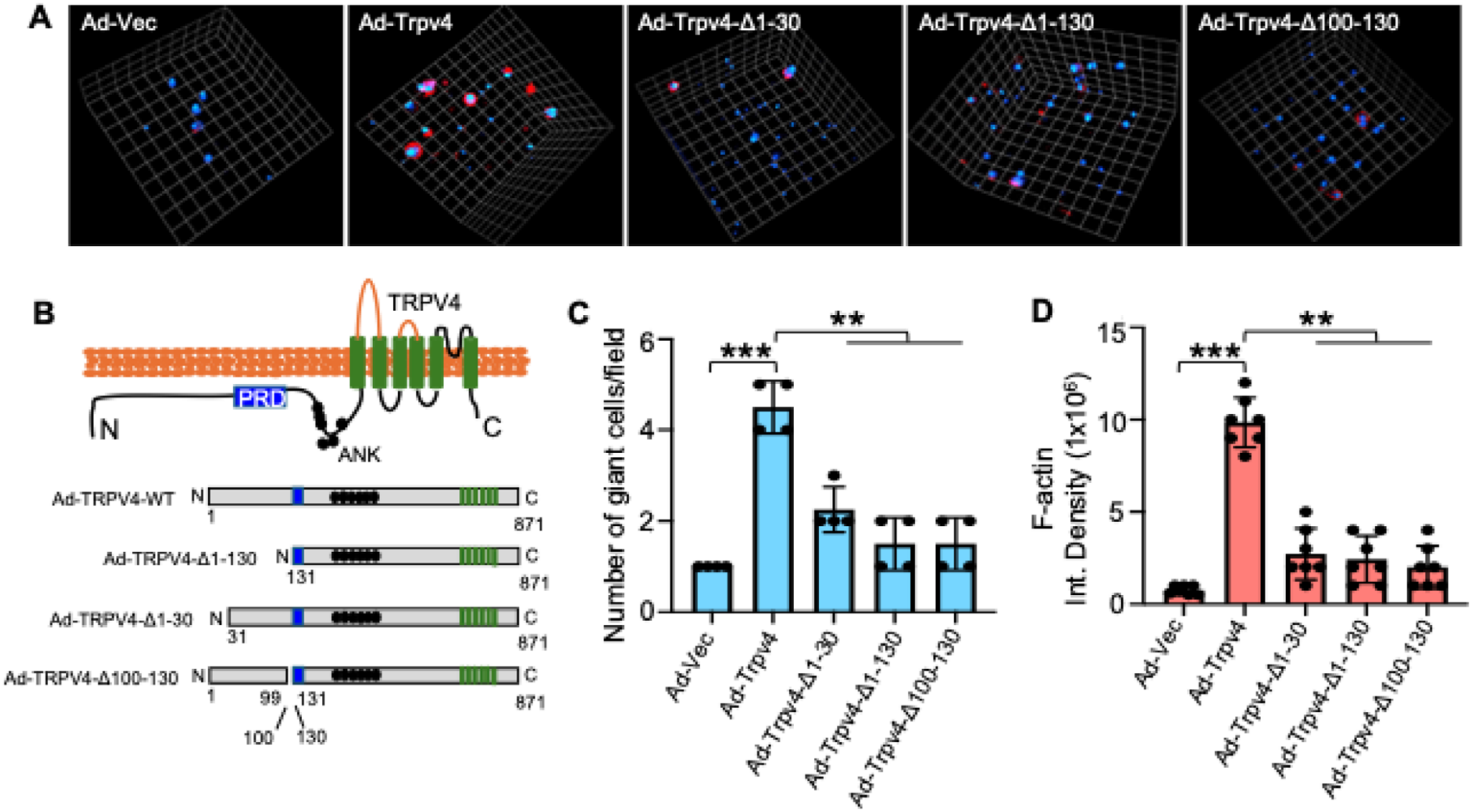
The N‐terminal 1–130 residues of TRPV4 are crucial for FBGC formation in response to mechanical stimulation in 3D microcapsules. A) Representative spinning‐disk confocal images of multinucleated FBGCs and F‐actin formation in TRPV4 KO BMDMs encapsulated in stiff 3D alginate‐collagen microcapsules following transfection with Ad‐Vec (control), Ad‐Trpv4, Ad‐Trpv4‐Δ1‐30, Ad‐Trpv4‐Δ1‐130, and Ad‐Trpv4‐Δ100‐130 constructs. Nuclei are stained with DAPI (blue), and F‐actin is stained with phalloidin (red). B) The schematic diagram shows the full‐length and mutant TRPV4 proteins. C) Quantification of the number of giant cells per field and D) quantification of F‐actin levels from the experiment in (A). Data are presented as the mean from three biological replicates, with 4–7 images analyzed per group; statistical analysis was performed using one‐way ANOVA. **p ≤ 0.01, and ***p ≤ 0.001.

To investigate the importance of N‐terminal residues of TRPV4 in FBGC formation within a 3D model, we utilized three TRPV4 N‐terminal deletion mutants (Ad‐Trpv4‐Δ1‐30, Ad‐Trpv4‐Δ1‐130, and Ad‐Trpv4‐Δ100‐130), developed using an adenoviral expression system. We transfected TRPV4 KO BMDMs with these mutants, as well as with an empty adenovector (Ad‐Vec) and full‐length WT‐TRPV4 (Ad‐Trpv4) and assessed FBGC formation within stiff microcapsules. Ten days post‐culture, spinning disk confocal microscopy was employed to visualize the BMDMs in their 3D environment inside the microcapsules. Microcapsules expressing Ad‐TRPV4 exhibited a threefold increase in FBGC formation and a tenfold increase in F‐actin generation (**Figure** **5**A–D) compared to the empty vector. In contrast, the absence of N‐terminal residues 1–30, 1–130, and 100–130 in TRPV4 led to a significant reduction in both FBGC formation and F‐actin generation (Figure 5A–D). These results highlight the critical role of the N‐terminal 1–130 residues of TRPV4 in FBGC formation in response to stiff microcapsules.

### 2.5 Expression of Inflammatory, Fibrotic, and Mechanosensitive Genes is Regulated by TRPV4 within 3D Microcapsules

**Figure 6:**
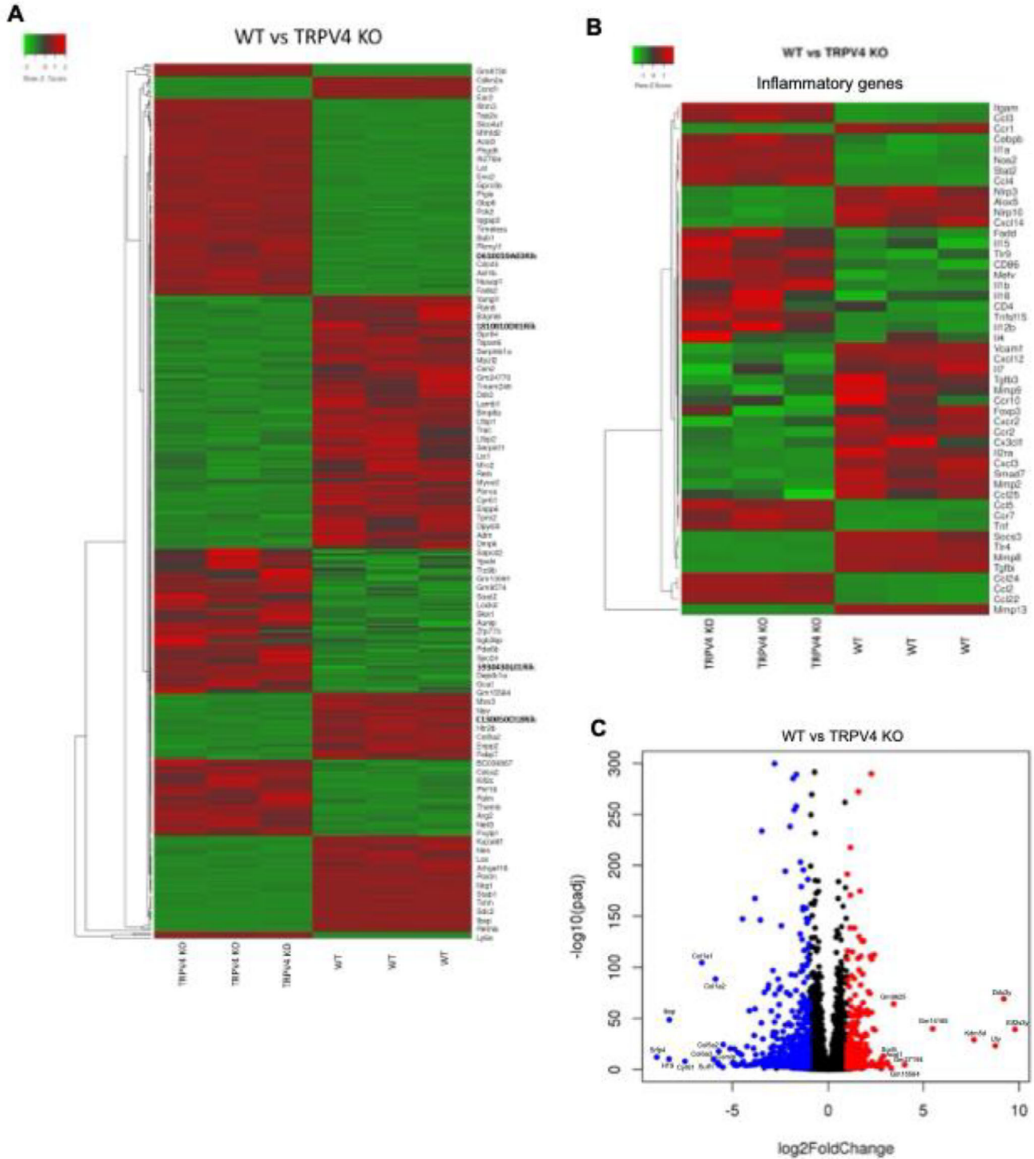
Bulk RNA sequencing (RNA‐seq) of WT and TRPV4 KO BMDMs encapsulated in 3D stiff microcapsules reveals differential gene expression. Bulk RNA‐seq analysis was conducted using RNA isolated from 3D alginate‐collagen microcapsules (2% alginate) encapsulating WT and TRPV4 KO BMDMs, following a 10‐day treatment with IL4 and GMCSF (25 ng mL^−1^). (A) Heatmap displaying the expression levels of the top 200 most upregulated and 200 most downregulated DEGs. TRPV4 modulates the expression of inflammatory genes in 3D microcapsules. B) Heatmap depicting the expression of 49 identified inflammatory genes. C) Volcano plot illustrating the differentially expressed genes (DEGs), with red dots representing upregulated DEGs and blue dots representing downregulated DEGs.

To investigate the effect of TRPV4 on the expression of genes related to FBR, we conducted bulk RNA sequencing (RNA‐seq) using stiff microcapsules embedded with WT and TRPV4 KO BMDMs. This analysis identified 1143 differentially expressed genes (DEGs) with a *p*‐value <0.05 and a log2 fold change of ±1, including 393 upregulated and 750 downregulated genes in TRPV4 KO BMDMs compared to WT, as illustrated in **Figure** **6**A–C. Heatmaps of the top 200 upregulated and downregulated DEGs were generated using the Heatmapper tool (Figure 6A).^[^ ^57^ ^]^

**Figure 7:**
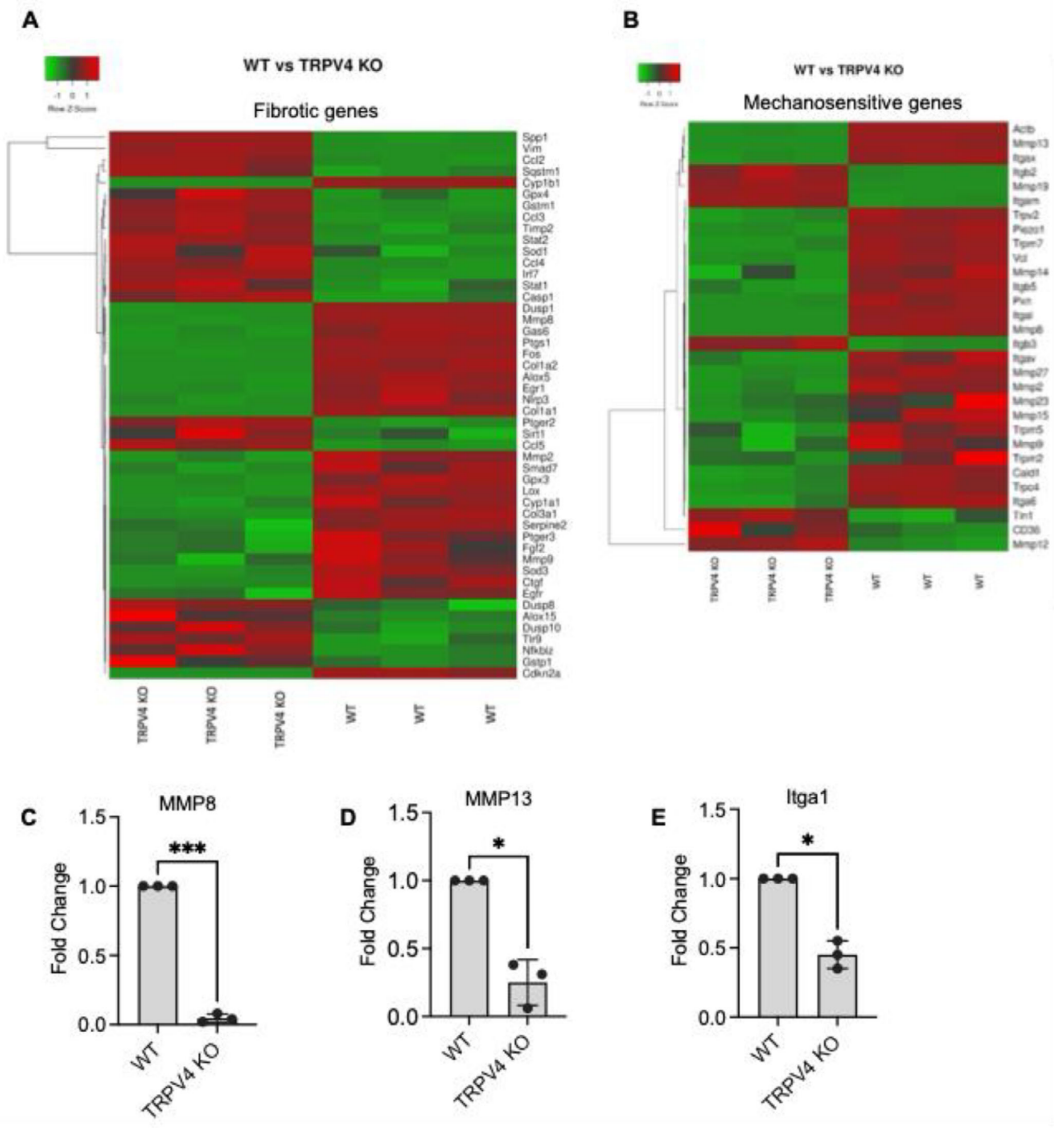
TRPV4 influences the expression of fibrotic and mechanosensitive genes in 3D microcapsules. A) Heatmap showing the expression of 48 identified fibrotic genes. B) Heatmap displaying the expression of 30 identified mechanosensitive genes. C–E) qRT‐PCR data shows expression of MMP8, MMP13, and Itga1 in WT and TRPV4 KO macrophages grown inside 2% 3D microcapsules for 10‐day in the presence of IL4 and GMCSF (25 ng mL^−1^). Statistical analysis was performed using one‐way ANOVA. *p ≤ 0.05, and ***p ≤ 0.001.

**Figure 8:**
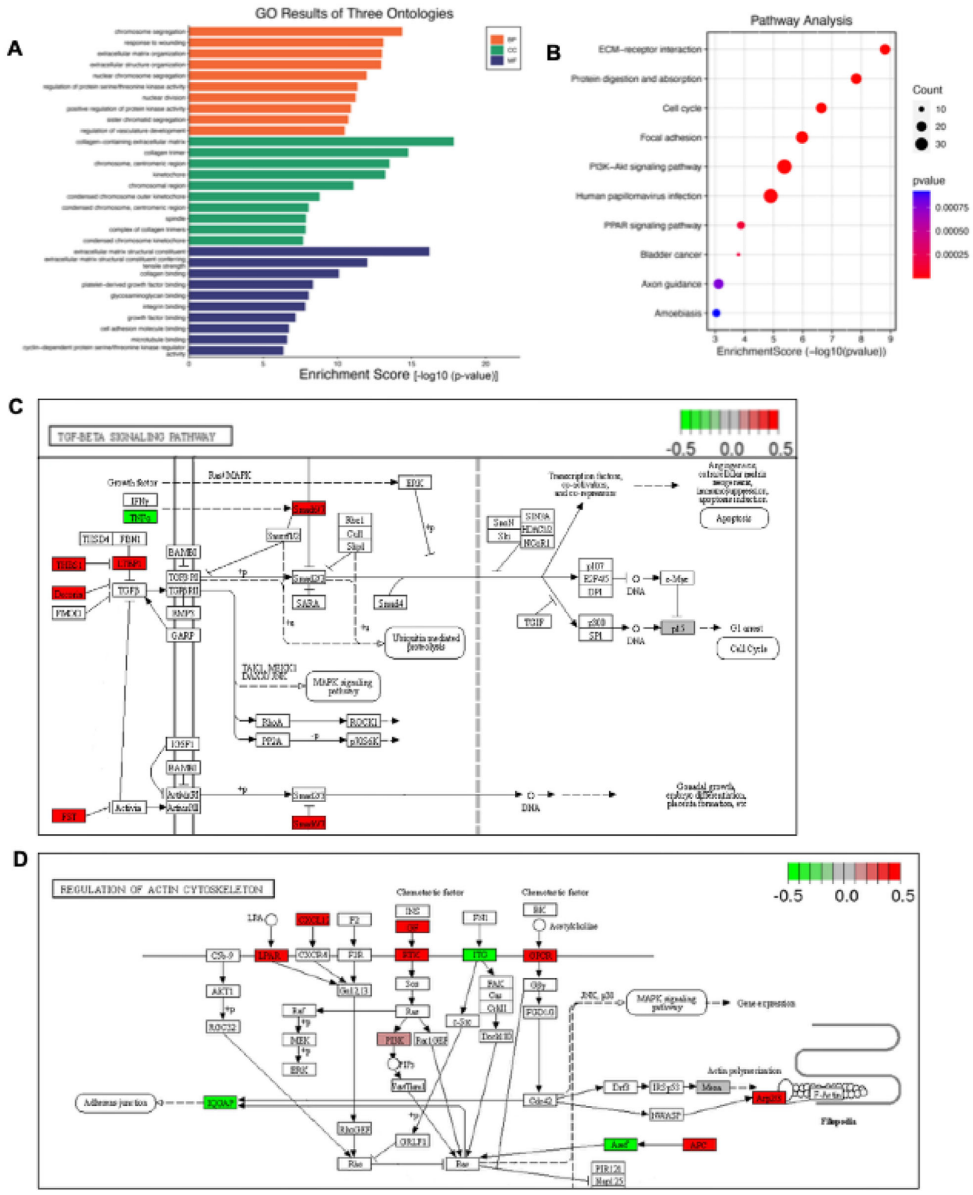
Gene ontology (GO) enrichment analysis of DEGs. A) Graphical representation of the top ten significant GO terms related to biological processes (BP), cellular components (CC), and molecular functions (MF). B) Bubble plot showcasing the KEGG pathway enrichment analysis, with the x‐axis indicating the enrichment score of the DEGs and the y‐axis representing the KEGG pathway names. The bubble size corresponds to the number of genes involved in each KEGG pathway, and the color scale denotes p‐value thresholds. C,D) KEGG pathway diagrams illustrating the dynamic interactions among the associated DEGs: (C) TGF‐β signaling pathway and (D) Regulation of actin cytoskeleton. Bright red represents the most upregulated, and bright green the most downregulated genes in TRPV4 KO compared to WT.

Furthermore, we identified DEGs associated with inflammatory, fibrotic, and mechanosensitive pathways, revealing 49 inflammatory DEGs, 48 fibrotic DEGs, and 30 mechanosensitive DEGs that were differentially expressed between WT and TRPV4 KO BMDMs (Figures 6B,C and **7**A,B; Figures S3 and S4, Supporting Information). Notably, there was substantial decrease in the expression of MMP2, MMP8, MMP9, MMP13, MMP15, TLR4, CTGF, Col1a1, Col1a2, Col3a1, Vinculin, Piezo1, TRPM7, TRPV2, and integrin subunit genes in TRPV4 KO BMDMs compared to WT (Figures 6B,C and 7A–E), suggesting a proinflammatory and fibrotic role by TRPV4 in BMDMs grown in stiff 3D microcapsules. Subsequently, we performed gene ontology (GO) enrichment analysis on the 1143 DEGs, which highlighted the top 10 enriched GO terms related to biological processes (BP), cellular components (CC), and molecular functions (MF) (**Figure** **8**A). This analysis underscored a variety of cellular responses critical in FBR development and progression, including cell adhesion molecule binding, integrin binding, response to wounding, extracellular matrix organization, and collagen binding. Pathway analysis using enrichment scores revealed the involvement of KEGG pathways associated with fibrotic responses, including the ECM‐receptor interaction, PI3K‐AKT signaling pathway, and focal adhesion pathways (Figure 8B–D). Collectively, these results indicate that TRPV4 regulates a gene expression signature that coordinates mechanosensation, cytoskeleton restructuring, and macrophage adhesion, all of which are essential for FBGC formation and development of the FBR.

## 3 Discussion

Although significant advances have been made in macrophage mechanobiology using 2D cell culture, it fails to fully replicate the 3D architecture of tissues, which can influence cellular behavior, cell–cell and cell–matrix interactions, morphology, and function compared to in vivo conditions.^[^ ^8^, ^9^, ^10^, ^11^, ^17^, ^24^, ^26^, ^30^ ^]^ Consequently, 2D cultures have limited capacity to capture the complex interactions that occur in a 3D environment. Therefore, evaluating immune responses to biomaterials and devices in a 3D model is essential for understanding the mechanisms underlying the body's response to biomaterials and for developing new strategies to create biocompatible biomaterials.

We successfully developed a heterogeneous alginate‐collagen 3D cell culture system using microfluidic technology to create core–shell hydrogel microcapsules. By varying alginate concentrations, we engineered microcapsules with stiffness levels resembling normal and fibrotic tissues, confirmed by atomic force microscopy. BMDMs encapsulated in both soft and stiff microcapsules remained viable over 17 days, demonstrating the model's suitability for in vitro studies. This system provides a versatile platform to study FBGC formation in a tissue‐like environment, offering a more accurate method for understanding cellular responses to varying stiffness in comparison to traditional 2D cultures. Notably, our 3D alginate‐collagen microcapsule model accurately replicated in vivo findings, showing that stiff microcapsules promoted a significant increase in FBGC number and volume over time, especially with IL4 and GMCSF treatment.^[^ ^10^, ^26^, ^27^, ^30^ ^]^ By day 17, there was a threefold increase in FBGC size and a fourfold increase in number, validating our in vitro model.

Cytoskeletal remodeling including F‐actin generation governs numerous physiological processes including cell fusion and phagocytosis.^[^ ^58^, ^59^ ^]^ Our study demonstrates that TRPV4 is crucial for stiffness‐dependent FBGC formation and F‐actin generation in a 3D microcapsule model. Encapsulating WT and TRPV4 knockout BMDMs revealed that, in WT cells, stiff microcapsules led to a threefold increase in FBGC formation and a fourfold rise in F‐actin generation compared to soft microcapsules. TRPV4 knockout BMDMs showed reduced FBGC formation and F‐actin production, indicating TRPV4's role in these processes. IL4 and GMCSF treatment further amplified FBGC formation and F‐actin generation in stiff WT microcapsules but not in TRPV4 knockout cells, confirming TRPV4's importance. Additionally, three NH_2_‐terminal deletion mutants of varying lengths (Ad‐TRPV4‐Δ1‐30, Ad‐TRPV4‐Δ1‐130, and Ad‐TRPV4‐Δ100‐130) were tested to assess the mechanosensing function of these domains in FBGC formation within our 3D model. These three deletions were specifically selected based on prior studies in a different cell type, which demonstrated the functional importance of these regions in TRPV4 activation by biomechanical and soluble stimuli using 2D models.^[^ ^10^, ^60^ ^]^ N‐terminal TRPV4 deletion mutants demonstrated that the absence of residues 1–130 significantly reduced FBGC formation and F‐actin production, emphasizing these residues' critical role. In contrast, our 2D model previously revealed that residues 100–130 of TRPV4 are critical for FBGC formation, whereas truncation of residues 1–30 had no discernible effect on FBGC formation.^[^ ^10^ ^]^ Activation of the TRPV4 has been shown to require binding of phosphatidylinositol 4,5‐bisphosphate (PIP2) to a specific N‐terminal region (residues 121–125).^[^ ^60^ ^]^ Deletion of residues 100–130 disrupts this interaction and suppresses channel activation. However, it remains unclear whether PIP2–TRPV4 binding is similarly necessary for FBGC formation in this 3D model and in vivo. Together, these findings establish TRPV4's essential function in cytoskeletal remodeling and FBGC generation in response to mechanical and biochemical stimuli, validating this 3D model for studying FBGC formation.

Bulk RNA‐seq of stiff microcapsules with WT and TRPV4 knockout BMDMs identified 1143 differentially expressed genes, with 393 upregulated and 750 downregulated in TRPV4 knockout BMDMs. Key pathways affected included inflammatory, fibrotic, and mechanosensitive genes. Gene ontology enrichment and KEGG pathway analysis revealed alterations in cell adhesion, ECM organization, and fibrotic responses, underscoring TRPV4's regulatory role in FBR processes in 3D context. These results suggest that we have successfully developed a 3D hydrogel cell culture system using microfluidic technology to engineer alginate‐collagen microcapsules that replicate tissue stiffness and architecture. This study using this 3D system demonstrates that TRPV4 is crucial for stiffness‐sensitive FBGC formation in a 3D microenvironment, with its N‐terminal residues 1–130 playing a key role. TRPV4 also regulates genes linked to inflammation, fibrosis, and mechanosensitivity, emphasizing its importance in macrophage‐mediated FBR. Overall, our findings suggest that TRPV4 plays a critical role in regulating gene expression patterns that are essential for macrophage fusion and the FBR. RNA‐seq analysis revealed that deletion of TRPV4 in mice significantly downregulated a range of genes implicated in extracellular matrix remodeling, mechanotransduction, and macrophage activation. Notably, TRPV4 KO macrophages exhibited decreased expression of lysyl oxidase, an enzyme that catalyzes collagen cross‐linking and facilitates cell adhesion and migration.^[^ ^61^ ^]^ This reduction may impair the structural remodeling necessary for FBGC formation. Likewise, connective tissue growth factor, which supports osteoclast and macrophage fusion in fibrotic and inflammatory settings, was also diminished in TRPV4 KO cells, supporting a potential role for TRPV4 in macrophage fusion during FBR.^[^ ^62^ ^]^ A hallmark of FBR is excessive collagen deposition and TGF‐β signaling. In our 3D model, TRPV4 deletion led to lower expression of TGF‐β3, Col1a1, and Col3a1 genes, suggesting that TRPV4 contributes to fibrotic matrix remodeling via regulation of collagen synthesis pathways. In parallel, multiple integrin subunits (Itga6, Itgv, Itga1, Itgax, and Itgb5) were downregulated in TRPV4 KO macrophages. Since integrin‐mediated signaling is critical for cytoskeletal reorganization, migration, and stiffness sensing, this downregulation may impair the mechanical cues necessary for FBGC formation.^[^ ^63^, ^64^, ^65^ ^]^ Interestingly, Piezo1‐ a known mechanosensor in macrophages—was also downregulated in TRPV4 KO cells, pointing to a broader disruption of mechanotransduction pathways in the absence of TRPV4.^[^ ^66^ ^]^ The interaction between TRPV4 and Piezo1 may represent a cooperative mechanosensitive axis influencing macrophage behavior in 3D environments. Matrix metalloproteinases (MMPs), including MMP2, MMP8, and MMP9, were significantly reduced in TRPV4 KO macrophages. MMP9, in particular, has been shown to facilitate macrophage fusion. Reduced MMP9 and MMP13 expression in the absence of TRPV4 may hinder matrix degradation and cell–cell fusion processes essential for FBGC formation.^[^ ^67^ ^]^ Collectively, our data indicate that TRPV4 regulates a gene expression program that coordinates mechanosensation, matrix remodeling, and macrophage fusion, all of which are critical for FBGC formation and progression of the FBR.

Overall, the results of this study demonstrate that our novel 3D core–shell hydrogel model provides a more accurate representation of in vivo conditions for studying macrophage behavior and FBGC formation compared to commonly used conventional 2D culture systems. When comparing 2D, 3D, and in vivo models, we observed clear discrepancies in key metrics such as FBGC formation time, FBGC size, and nuclei per FBGC, with the 3D model closely mirroring in vivo outcomes. Specifically, FBGC formation was markedly faster in the 2D culture (6 days) but required significantly longer timeframes in the 3D hydrogel model (17 days) and in vivo (28 days). This extended timeline in the 3D and in vivo models likely reflects the more physiologically relevant mechanical and structural cues absent in 2D cultures.

The differences in FBGC size and nuclei per FBGC further underscore the limitations of 2D systems in replicating the complexity of in vivo environments. FBGCs in the 2D culture were significantly larger (70 µm) and contained a higher number of nuclei (26 nuclei) compared to both the 3D model (40 µm, 12 nuclei) and the in vivo scenario (50 µm, 10 nuclei). These observations suggest that the flat, rigid substrate of 2D cultures may induce exaggerated cell spreading and fusion, leading to larger, multinucleated cells that are not representative of physiological conditions. In contrast, the 3D hydrogel model appears to offer a more accurate mimic of the in vivo ECM, providing mechanical resistance and spatial confinement that regulate macrophage fusion and FBGC formation more effectively. While our results show that both 3D and in vivo systems differ significantly from conventional 2D culture with respect to nuclei per FBGC, FBGC size, and the timing of FBGC formation, they also reveal a key limitation of the 3D microcapsule model. Although nuclei per FBGC and FBGC size were comparable between the 3D and in vivo systems, FBGC formation occurred significantly faster in the 3D model. This discrepancy suggests that the macrophage‐only 3D system does not fully replicate the temporal dynamics of the in vivo foreign body response, likely due to the absence of critical cell–cell interactions. Incorporating additional cell types—such as endothelial cells and fibroblasts—into the 3D microcapsules may better mimic the complex multicellular environment of in vivo tissues. Furthermore, given that macrophage phenotypes extend beyond the classical M1/M2 dichotomy, future studies utilizing high‐resolution techniques such as single‐cell RNA sequencing will be essential to comprehensively characterize macrophage heterogeneity and evaluate the fidelity of the 3D system in capturing in vivo cellular diversity and activation states.

The improved alignment of the 3D hydrogel model with in vivo metrics can be attributed to its core–shell architecture, which better replicates the biomechanical properties of the dermal ECM encountered during the FBR. The core–shell design provides a heterogeneity of stiffness and porosity, facilitating macrophage mechanosensing and cytoskeletal remodeling in a manner that is more reflective of the in vivo tissue environment. This finding is consistent with previous studies highlighting the importance of substrate stiffness and dimensionality in regulating macrophage behavior and fusion dynamics.^[^ ^10^ ^]^

The ability of our 3D model to recapitulate key aspects of the FBR has important implications for preclinical research. Traditional 2D and 3D culture systems, while convenient and widely used, fail to capture the complex mechanical and biochemical interactions that occur in vivo. This discrepancy may lead to misleading conclusions about macrophage behavior, FBGC formation, and the efficacy of potential therapeutic interventions targeting the FBR. By contrast, our 3D hydrogel model offers a more physiologically relevant platform that could improve the predictive value of in vitro studies, reducing the reliance on animal models and enhancing the translational potential of preclinical findings. Despite advances, traditional 3D in vitro models for studying the FBR and FBGC formation have notable limitations. These models often lack the complexity of the in vivo tissue environment, including dynamic mechanical cues, spatial and biochemical heterogeneity, proper oxygen, nutrient, or cytokine gradients, and real‐time imaging capabilities. They typically focus on macrophages alone, omitting other key immune and stromal cells involved in the FBR. Most models are also avascular, limiting nutrient diffusion and immune cell recruitment, and they are poorly suited for studying chronic responses due to limited long‐term stability. Additionally, the biomaterials used often lack physiologically relevant surface features or degradation profiles. These limitations reduce the predictive power of current models, underscoring the need for more physiologically relevant, multicellular, and dynamically tunable 3D systems. Our 3D macrophage cell culture model provides a physiologically relevant platform to study the full range of FBRs by mimicking the complex mechanical and biochemical environment of tissue. By embedding macrophages in tunable hydrogels or encapsulating them with biomaterial particles, researchers can replicate early inflammatory responses, chronic activation, FBGC formation, and fibrosis. This model allows investigation of how material properties—such as stiffness, degradability, and surface chemistry—influence macrophage behavior, including cytokine production, fusion into FBGCs, and interactions with fibroblasts leading to fibrotic capsule formation. Through controlled manipulation of the microenvironment, this 3D model enables mechanistic insights into immune‐material interactions and serve as valuable tools for screening biomaterials and testing interventions to mitigate FBR.

In summary, our findings demonstrate the 3D core–shell hydrogel model as a superior platform for replicating in vivo macrophage responses and FBGC formation, closely mirroring key metrics such as formation time, size, and nuclei count. This model revealed that hydrogel stiffness as well as TRPV4 activity, significantly influence FBGC formation and F‐actin production, with effects enhanced by IL4 and GMCSF priming. RNA‐seq analysis highlighted TRPV4's role in regulating inflammatory, fibrotic, and mechanosensitive gene expression, offering valuable insights into macrophage behavior and FBR mechanisms. This model represents a significant advance over commonly used conventional 2D cultures, providing a valuable tool for studying the mechanisms underlying the FBR and evaluating novel therapeutic strategies. Future work should explore the potential of this 3D model in various contexts of immune modulation and implant biocompatibility, as well as its applicability across different cell types and tissue environments.

## 4 Experimental Section

### Materials

Dulbecco's Modified Eagle's Medium (DMEM), fetal bovine serum (FBS), antibiotic‐antimycotic, and other related cell culture reagents were obtained from Gibco. Mouse recombinant IL4, GMCSF, and M‐CSF were sourced from R&D Systems. Alexa Fluor 594 Phalloidin was acquired from Thermo Fisher Scientific. Sodium alginate (A1112) was purchased from Sigma (St. Louis, MO), followed by purification through a sequential process involving washing with chloroform and 1% (w/v) charcoal, and then freeze‐dried to remove water. GSK2193874 (GSK219) was purchased from Sigma. Collagen type I (354249) was obtained from Corning (NY). For the microfluidic device connections, Masterflex Transfer Tubing, including the MFLX 95802‐00 and MFLX 06417‐11 tubing, was purchased from VWR (Radnor, PA). Slip Tip syringes, both 1.0 and 10.0 mL, were ordered from Becton, Dickinson (BD, Franklin Lakes, NJ). SU‐8 (SU‐8 2050) and SU‐8 developer were sourced from Kayaku Advanced Materials (Westborough, MA). Additionally, silicon wafers were procured from University Wafer (South Boston, MA).

### Animal

The laboratory acquired wild‐type (WT) C57BL/6 mice from Charles River Laboratories, USA. The TRPV4 knockout mice on a C57BL/6 background were originally generated by M. Suzuki at Jichi Medical University, Tochigi, Japan. The mouse colony was maintained in a controlled environment with regulated humidity, temperature, and specific pathogen‐free conditions. All experimental procedures and animal protocols were approved by the Institutional Animal Care and Use Committee at the University of Maryland (protocol # 2167644‐2).

### In Vivo FBR Model and Histology

To induce the formation of a foreign body response in vivo, subcutaneous implantation of mixed cellulose ester discs was performed.^[^ ^10^ ^]^ After 28 days, the implants and surrounding skin tissue were surgically excised and embedded in OCT (Optimal Cutting Temperature) compound. Cryosections of 7–10 µm thickness were prepared using a cryostat and mounted on positively charged slides. The tissue sections were then fixed in 10% formalin and stained with Hematoxylin and Eosin (H&E) following the manufacturer's protocol.

### Fabrication of Microfluidic Device

The non‐planar microfluidic devices were fabricated using a soft lithography process with polydimethylsiloxane (PDMS) on a mold or master, which was created through photolithography techniques.^[^ ^45^, ^46^, ^47^, ^48^, ^49^ ^]^ The design of the microfluidic device was developed in AutoCAD (Autodesk, Mill Valley, CA), and a corresponding design mask was produced by CAD/Art Service (Brandon, OR). The mold preparation involved several steps: first, a 100 µm thick layer of SU‐8 2050 photoresist was spin‐coated onto a 4‐inch silicon wafer, followed by hot plate treatments at 65 and 95 °C. The wafer was then exposed to UV light through the patterned mask using a MA‐4 Mask Aligner (Karl Suss, Munich, Germany) to pattern the core channel. After post‐exposure baking, an additional 50 µm thick layer of SU‐8 2050 was applied, baked at 65 and 95 °C, and UV‐exposed to pattern the shell channel. Another 50 µm thick layer of SU‐8 2050 was applied, followed by the same baking and UV exposure process to pattern the oil channel. Finally, the wafer was developed using SU‐8 developer and underwent post‐baking at 120 °C. The specific spin speeds, baking parameters, and exposure energy were all determined according to the SU‐8 2050 datasheet provided by the manufacturer.

PDMS was prepared by mixing the base and curing agent in a 10:1 (w/w) ratio. The top and bottom parts of the microfluidic device were created by pouring PDMS over the mold, followed by degassing and baking at 75 °C for 2 h. The PDMS with the designed pattern was carefully peeled from the mold, plasma‐treated using a PDC‐32G plasma cleaner (Harrick Plasma, Ithaca, NY) at 18 W and 27 Pa for 2 min, and aligned under a Zeiss Primovert microscope. The devices were then baked at 75 °C for 3 h before further use.

### Microencapsulation of BMDMs

Microencapsulation of bone marrow‐derived macrophages (BMDMs) in core–shell hydrogel microcapsules was conducted by using a microfluidic device, as illustrated in Figure 1A,B. The device has separate channels for flowing the core fluid, shell fluid, and oil. The core fluid was prepared by mixing 200 µL of neutralized collagen type I (6.0 mg mL^−1^) with 200 µL of 4% sodium alginate solution.^[^ ^45^, ^46^, ^47^, ^48^, ^49^ ^]^ This mixture was then used to suspend BMDMs at the desired concentration before being transferred to a 1 mL syringe. The shell fluid was consisted of a 2% sodium alginate solution. For the oil channel fluid, an emulsion was prepared by mixing mineral oil with 1 g mL^−1^ aqueous calcium chloride solution in a 5:1 volume ratio, supplemented with 1.5% Span 80. This emulsion was sonicated for 1 min using a Branson 450 digital sonifier (Emerson, St. Louis, MO). All fluids were introduced into the microfluidic device using a syringe pump (Harvard Apparatus, Holliston, MA) at flow rates of 200 µL h^−1^ for the core channel, 500 µL h^−1^ for the shell channel, and 5.0 mL h^−1^ for the oil channel. The resulting microcapsules were collected in a 50‐mL tube containing 0.25 m D‐mannitol solution with 10 mm HEPES (pH 7.4). After removing the mineral oil, the microcapsules were washed with 0.25 m D‐mannitol solution, gelled in 200 mm calcium chloride solution for 10 min, and then washed twice more with 0.25 m D‐mannitol solution before being resuspended in complete DMEM medium. This medium, containing 10% FBS, was used to culture the microcapsules in an incubator at 37 °C with 5% CO_2_.

The procedure for producing microcapsules with different core materials or cell numbers was identical to the steps described above, except for the preparation of the core fluid. In these cases, instead of mixing 200 µL of neutralized collagen type I (6.0 mg mL^−1^) with 200 µL of a 4% sodium alginate solution, 200 µL of neutralized collagen type I (6.0 mg mL^−1^) was combined with 200 µL of a 1% sodium alginate solution to resuspend the different cell counts.

### Generation of FBGC In Vitro in 3D Alginate‐Collagen Microcapsules

Bone marrow was isolated from the femurs of wild‐type (WT) and TRPV4 null mice and cultured in DMEM supplemented with 10% FBS and 25 ng mL^−1^ MCSF. The cultures were incubated at 37 °C with 5% CO_2_ for 7–8 days to generate mature BMDMs.^[^ ^10^, ^11^, ^15^ ^]^ These mature BMDMs were then encapsulated within sodium alginate beads of varying core materials and cell numbers to investigate the formation of FBGCs using an IL4 plus GMCSF cocktail (25 ng mL^−1^), administered every 48 h. F‐actin was stained using Alexa Fluor 594‐labeled phalloidin, and nuclei were stained with DAPI. Fluorescent imaging was performed at 10X magnification using a Zeiss Axio Observer Z1 microscope.

### Live/Dead Staining

After various incubation periods, the microcapsules were collected and stained with Calcein AM (Corning, Inc., Corning, NY) and propidium iodide (PI) (Invitrogen, Carlsbad, CA) for 15 min. Following three washes with complete DMEM medium, the samples were visualized using a 10X phase lens on a fluorescence microscope (Zeiss LSM 710).

### FBGC Formation from BMDM and Adenovirus Transfection

Bone marrow isolated from the femurs of TRPV4 null mice was cultured in DMEM supplemented with 10% FBS and 25 ng mL^−1^ MCSF and incubated at 37 °C with 5% CO_2_ for 7–8 days to generate mature BMDMs. These mature BMDMs were then transfected with Ad‐TRPV4 constructs or empty vector controls (working concentration: 10⁵ plaque‐forming units mL^−1^) for 48 h before being encapsulated in 2% sodium alginate beads.^[^ ^10^ ^]^ The beads were treated with an IL4 and GMCSF cocktail (25 ng mL^−1^) every 48 h for 10 days. Afterward, the sodium alginate beads were fixed with 8% paraformaldehyde and stained with Alexa Fluor 594‐labeled phalloidin for F‐actin and DAPI for nuclei.

### Spinning Disk Confocal Microscopy

Bone marrow cells were harvested from the femurs of wild‐type mice and cultured in DMEM supplemented with 10% fetal bovine serum. To promote macrophage differentiation, cells were treated with macrophage colony‐stimulating factor (M‐CSF, 25 ng mL^−1^) for 8 days. Mature macrophages were then encapsulated within 2% stiff alginate‐collagen microcapsules and treated with IL4 and GM‐CSF (25 ng mL^−1^) every 48 h for 10 days. In parallel, microcapsules were exposed to GSK2193874 at concentrations of 2 and 10 µm on the same schedule. At the end of the treatment period, microcapsules were fixed with 4% paraformaldehyde and stained with Alexa Fluor 594‐conjugated phalloidin to label F‐actin and with DAPI to visualize nuclei. To generate fluorescent 3D images of the FBGCs, a PerkinElmer spinning‐disk confocal microscope with 20X magnification was used. Data collection was performed using the PerkinElmer Velocity software (version 6.4.0), which is equipped with a Hamamatsu ImagEM X2 EM‐CCD camera (C9100‐23B). Images were acquired using 405 nm (DAPI) and 561 nm (F‐actin) wavelength lasers, with Z‐slices captured at every 1 µm interval.

### RNA‐Sequencing and Gene Enrichment Analysis

RNA samples were extracted from BMDMs cultured within 2% sodium alginate beads, using DMEM supplemented with 10% FBS and an IL‐4 plus GM‐CSF (25 ng mL^−1^) cocktail over 10 days. RNA sequencing was outsourced to Azenta (New Jersey, USA). Genes with a padj‐value <0.05, log_2_FoldChange <−1, or log_2_FoldChange > 1 were identified as differentially expressed genes (DEGs). These DEGs were used to generate heatmaps via the Heatmapper web tool (http://heatmapper.ca/).^[^ ^50^, ^51^ ^]^ Additionally, Gene Ontology (GO) function analysis, KEGG pathway analysis, and KEGG pathway enrichment analysis were conducted using the free web tool iDEP (version 0.96)^[^ ^50^ ^]^ and Pathview tools.^[^ ^52^, ^53^ ^]^

### qRT‐PCR Analysis

Total RNA was isolated from WT and TRPV4 KO macrophages grown inside 2% 3D microcapsules for 10‐day in the presence of IL4 and GMCSF (25 ng mL^−1^), following the instructions provided in the Qiagen kit. Subsequently, a qRT‐PCR was conducted using the One‐Step Quantitative Reverse Transcription PCR kit from Bio‐Rad. To determine the gene expression levels of each gene relative to the mRNA levels of GAPDH, the comparative C_T_ method was employed as outlined in the Bio‐Rad qRT‐PCR user manual.

### Atomic Force Microscopy

Atomic force microscopy (AFM) was conducted using the JPK NanoWizard 4 AFM (Bruker Nano GmbH, Berlin, Germany) to measure the stiffness of alginate monolayers with varying alginate concentrations (0.5% and 2%) adhered to the surface of a 35 mm glass Petri dish.^[^ ^10^ ^]^ The alginate layer was kept submerged in sterile PBS during measurements, which were performed directly on the dish using contact mode force spectroscopy to record individual force–distance (F‐D) curves. CP‐qp‐CONT‐BSG colloidal probes (sQube) with a nominal resonance frequency of 30 kHz in air, a spring constant of 0.1 N m^−1^, and an attached 10 µm diameter borosilicate glass sphere were used for the measurements. The cantilever spring constant and photodiode sensitivity were calibrated individually before each experiment. A relative set point of 2 nN, an extended speed of 10 µm s^−1^, and a Z length of 12 µm were chosen to obtain suitable F‐D curves. Thirty F–D curves were generated across different areas on the gels and processed using the Hertz contact mechanics model for spherical probes to calculate the Young's modulus (E). Bar plots of the Young's modulus (E) for both the 0.5% and 2% alginate gels were created using GraphPad Prism version 10.

### Rheological Characterization of Core Materials

Rheological properties of the core material (mixture of collagen I and alginate) inside 3D alginate‐collagen microcapsules (8 mm × 3 mm, diameter × height) were tested using an 8‐mm‐diameter parallel plate at a 1 mm gap on a stress‐controlled TA Instrument (New Castle, DE) ARES‐G2 Rheometer. All samples were tested at 37 °C. Frequency sweeps were performed at a strain of 1%.

### Statistical Analysis

All the data were acquired in triplicate unless specified otherwise. Data were presented as mean ± standard deviation (SD) values. Statistical significance between sample groups was determined by Student's t‐test or one‐way ANOVA, and represented as follows: *p ≤ 0.05, **p ≤ 0.01, ***p ≤ 0.001, and ****p ≤ 0.0001. Prism 10 was used for statistical analysis.

## Conflict of Interest

The authors declare no conflict of interest.

## Author Contributions

M.M. and W.O. contributed equally to this work. S.O.R. and X.H. conceived the study, designed and analyzed data, and edited the manuscript. M.M., and W.O. performed the experiments. S.O.R. and M.M. wrote the manuscript. J.S.B. and X.Z. edited the manuscript. Bidisha Dutta helped in AFM study. All authors reviewed and approved the final content of the manuscript.

## Supporting information



Supporting Information

## Data Availability

The data that support the findings of this study are available from the corresponding author upon reasonable request.
