# Supplementary material for: A Novel Core–Shell Hydrogel 3D Model for Studying Macrophage Mechanosensing and Foreign Body Giant Cell Formation
Source: Adv Healthc Mater. 2025 Sep 20;15(3):e01614. doi: 10.1002/adhm.202501614 (PMC12817113; doi:10.1002/adhm.202501614)
Supplement: Supplementary file 1 — Supporting Information [file ADHM-15-0-s001.pdf]

ADVANCED HEALTHCARE MATERIALS

Supporting Information

for *Adv. Healthcare Mater.*, DOI 10.1002/adhm.202501614

A Novel Core–Shell Hydrogel 3D Model for Studying Macrophage Mechanosensing and Foreign Body Giant Cell Formation

Manisha Mahanty, Wenquan Ou, Xiaoping Zhu, Jonathan S Bromberg, Xiaoming He and Shaik O. Rahaman**

A Novel Core-Shell Hydrogel 3D Model for Studying Macrophage Mechanosensing and Foreign Body Giant Cell Formation

Manisha Mahanty[1#], Wenquan Ou[2#], Xiaoping Zhu[3], Jonathan S Bromberg[4], Xiaoming He*[2], Shaik O. Rahaman*[1]

[1]University of Maryland, Department of Nutrition and Food Science, College Park, MD 20742
[2]University of Maryland, Fischell Department of Bioengineering, College Park, MD 20742
[3]University of Maryland, Department of Veterinary Medicine, College Park, MD 20742
[4]University of Maryland School of Medicine, Baltimore, MD 21201

Running title: 3D model for giant cell formation

*To whom correspondence should be addressed: Shaik O. Rahaman, University of Maryland, Department of Nutrition and Food Science, College Park, MD 20742, Tel: (301) 405-2940, Email: srahaman@umd.edu;
Xiaoming He, University of Maryland, Fischell Department of Bioengineering, College Park, MD 20742, Tel: (301) 405-7946, Email: shawnhe@umd.edu

# Equal contribution

Supplemental Figure 1

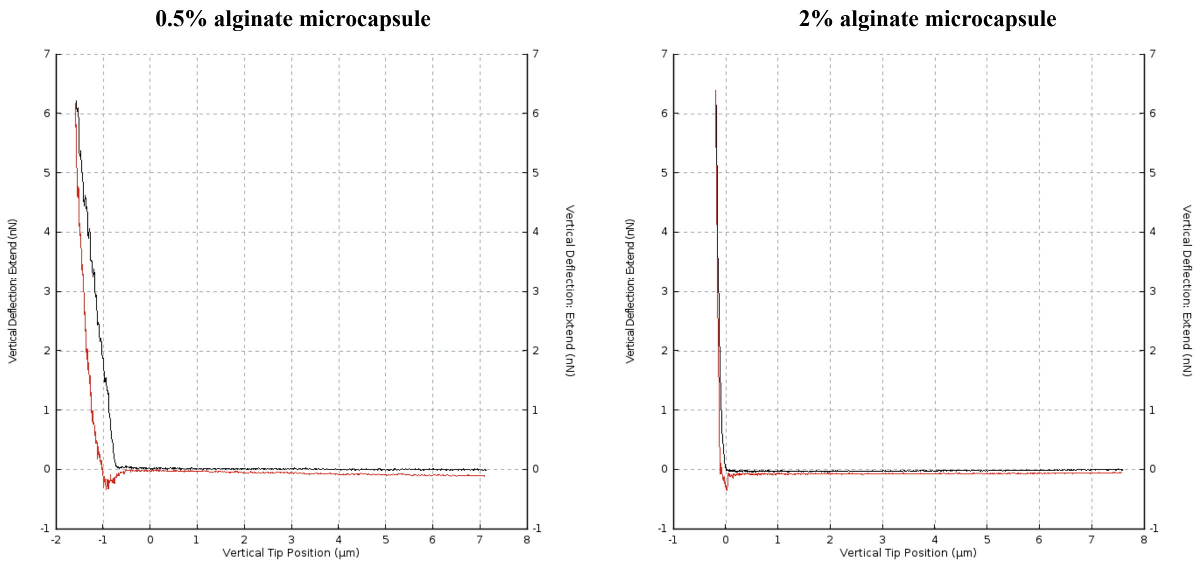

Supplemental Figure 1: Representative force-displacement curves obtained on 0.5% and 2% collagen-alginate microcapsules.

Supplemental Figure 2

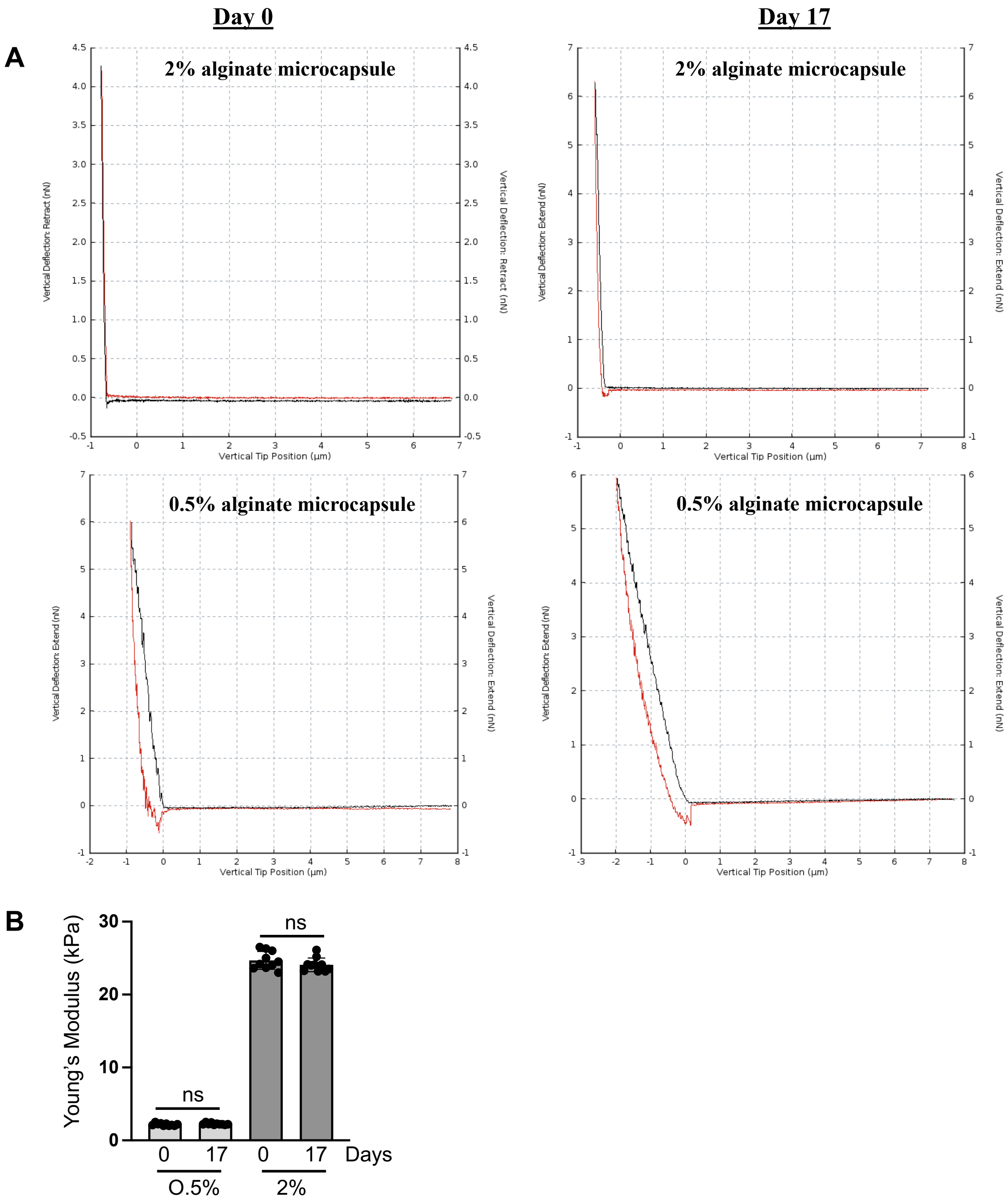

Supplemental Figure 2: A). Representative force-displacement curves obtained on 0.5% and 2% collagen-alginate microcapsules at day 0 and 17. (B). Bar graphs show quantification of stiffness from A. ns: not significant, student t-test.

Supplemental Figure 3

49 Inflammatory genes (WT vs. TRPV4 KO)				
**Gene**	**Description**	**Fold Change**	**P-value**	**Biological Function**
Alox5	Arachidonate 5-Lipoxygenase	5.56	1.8E-83	Inflammatory response
Cxcl12	C-X-C Motif Chemokine Ligand 12	5.05	2E-07	Regulation of monocyte chemotaxis, calcium ion transport
Cxcl3	C-X-C Motif Chemokine Ligand 3	5.01	7E-11	Chemokine-mediated signaling pathway, positive regulation of cytosolic calcium ion concentration
Cxcl14	C-X-C Motif Chemokine Ligand 14	4.71	1E-45	Cell-cell signaling
Il2ra	Interleukin 2 Receptor Alpha	4.67	3E-09	Immune response, Notch signaling pathway
Mmp8	Matrix Metalloproteinase 8	4.29	0E+00	Collagen catabolic process
Mmp2	Matrix Metalloproteinase 2	3.58	2E-14	Positive regulation of vascular SMC proliferation, cell migration
Nlrp10	Nucleotide-Binding Domain, Leucine-Rich Repeat Family, Pyrin Domain Containing 10	3.58	9E-27	Regulation of T-helper 1 type immune response, innate immunity
Smad7	SMAD Family Member 7	3.53	1E-14	Adherence junction assembly, protein ubiquitination
Mmp9	Matrix Metalloproteinase 9	3.42	3E-03	Negative regulation of fibroblast proliferation
Vcam1	Vascular Cell Adhesion Molecule 1	3.04	8E-05	Cell adhesion, cell chemotaxis
Ccr2	C-C Chemokine Receptor Type 2	2.13	2E-04	Positive regulation of inflammatory response
Cx3cl1	C-X3-C Motif Chemokine Ligand 1	2.11	7E-04	Angiogenesis, leukocyte migration
Tgfb3	Transforming Growth Factor Beta 3	1.98	5E-02	Positive regulation of collagen biosynthetic process
Nlrp3	Nucleotide-Binding Domain, Leucine-Rich Repeat Family, Pyrin Domain Containing 3	1.97	5E-19	Negative regulation of NLRP3 inflammasome complex assembly
Foxp3	Forkhead Box P3	1.75	1E-01	Interleukin-4 production, cell proliferation
Cxcr2	C-X-C Chemokine Receptor Type 2	1.73	4E-03	Positive regulation of cytosolic calcium ion concentration
Tgfbi	Transforming Growth Factor Beta-Induced	1.71	2E-74	Cell adhesion, extracellular matrix organization
Ccr10	C-C Chemokine Receptor Type 10	1.70	2E-01	Positive regulation of cytosolic calcium ion concentration
Mmp13	Matrix Metalloproteinase 13	1.63	1E-294	Collagen catabolic process
Tlr4	Toll-Like Receptor 4	1.59	5E-43	Positive regulation of NLRP3 inflammasome complex assembly, positive regulation of stress-activated MAPK cascade
Ccl25	C-C Chemokine Ligand 25	1.56	7E-03	Proinflammatory cytokine
Il7	Interleukin 7	1.52	8E-02	Positive regulation of chemokine production, immune response
Ccr1	C-C Chemokine Receptor Type 1	1.49	2E-69	Response to wounding, cell adhesion
Socs3	Suppressor of Cytokine Signaling 3	1.36	5E-19	Negative regulation of inflammatory response, negative regulation of receptor signaling pathway via JAK-STAT
Ccl4	C-C Chemokine Ligand 4	0.72	2E-17	Inflammatory response
Stat2	Signal Transducer and Activator of Transcription 2	0.69	4E-26	Receptor signaling pathway via JAK-STAT, protein phosphorylation
Ccl3	C-C Chemokine Ligand 3	0.68	2E-44	Calcium-mediated signaling, cellular calcium ion homeostasis
Cd4	Cluster of Differentiation 4	0.68	8E-02	Cell adhesion, regulation of calcium ion transport
Il1b	Interleukin 1 Beta	0.67	4E-03	Positive regulation of interferon-gamma production, positive regulation of prostaglandin biosynthetic process
Il15	Interleukin 15	0.67	6E-04	Inflammatory response
Il1a	Interleukin 1 Alpha	0.65	2E-21	Cytokine-mediated signaling pathway
Ccl22	Chemokine (C-C motif) Ligand 22	0.65	4E-97	Positive regulation of ERK1 and ERK2 cascade
Itgam	Integrin Alpha M	0.64	1E-69	Cell-cell adhesion via plasma-membrane adhesion molecules
Il18	Interleukin 18	0.63	7E-02	Cell adhesion, foam cell differentiation
Cebpb	CCAAT/enhancer-binding protein beta	0.62	6E-19	Inflammatory response, cellular response to interleukin 1
Ccl2	Chemokine (C-C motif) Ligand 2	0.61	1E-142	Cytokine-mediated signaling pathway, macrophage chemotaxis
Cd86	Cluster of Differentiation 86	0.60	9E-08	Response to interferon-gamma, cell-cell signaling
Fadd	Fas-Associated Death Domain Protein	0.60	1E-06	Positive regulation of macrophage differentiation
Il4	Interleukin 4	0.60	3E-01	Cytokine-mediated signaling pathway
Tlr9	Toll-Like Receptor 9	0.58	6E-06	Positive regulation of inflammatory response
Ccl24	Chemokine (C-C motif) Ligand 24	0.54	4E-265	Inflammatory processes
Ccr7	C-C Chemokine Receptor Type 7	0.53	3E-18	Release of sequestered calcium ion into cytosol, positive regulation of cell-matrix adhesion
Nos2	Nitric Oxide Synthase 2	0.50	7E-58	Cytokine production involved in inflammatory response
Mefv	Mediterranean Fever Gene	0.47	1E-05	Positive regulation of autophagy, negative regulation of cytokine production involved in inflammatory response
Tnf	Tumor Necrosis Factor	0.46	4E-32	Calcium-mediated signaling, regulation of cell proliferation
Ccl5	C-C Chemokine Ligand 5	0.37	2E-36	Positive regulation of homotypic cell-cell adhesion, positive regulation of gene expression
Il12b	Interleukin 12 Subunit Beta	0.16	5E-07	Positive regulation of NK T cell proliferation, positive regulation of interferon-gamma production
Tnfsf15	Tumor Necrosis Factor Superfamily Member 15	0.15	3E-09	Signal transduction, activation of NF-kappa b

Supplemental Figure 3: Tables detailing fold changes, p-values, descriptions, and biological functions of differentially expressed inflammatory genes in WT and TRPV4 KO BMDMs grown inside 2% 3D microcapsules for 10-day in the presence of IL4 and GMCSF (25 ng/ml).

Supplemental Figure 4

A

48 Fibrotic genes (WT vs. TRPV4 KO)				
Gene	Description	Fold change	P-value	Biological function
Col1a1	Collagen Type I Alpha 1 Chain	96.674	1.9988E-107	Collagen biosynthetic process, wound healing
Col1a2	Collagen Type I Alpha 2 Chain	58.960	2.99009E-91	Collagen fibril organization
Ctgf	Connective Tissue Growth Factor	29.626	1.49468E-07	Cell-cell signaling, cell adhesion
Col3a1	Collagen Type III Alpha 1 Chain	24.746	1.96302E-17	Negative regulation of immune response, transforming growth factor beta receptor signaling pathway
Cdkn2a	Cyclin-Dependent Kinase Inhibitor 2A	11.070	5.4822E-237	Negative regulation of cell growth, protein binding
Sod3	Superoxide Dismutase 3	8.514	7.31883E-08	Response to hypoxia
Cyp1a1	Cytochrome P450 1A1	8.028	2.21525E-12	Oxidation-reduction process
Alox5	Arachidonate 5-Lipoxygenase	5.564	1.83362E-83	Inflammatory response, acute inflammatory response
Lox	Lysyl Oxidase	5.115	2.63748E-39	Collagen fibril organization, regulation of cell-substrate adhesion
Mmp8	Matrix Metalloproteinase 8	4.292	0.0000001	Collagen catabolic process, proteolysis
Cyp1b1	Cytochrome P450 1B1	3.877	0.0000001	Blood vessel morphogenesis, negative regulation of cell migration
Mmp2	Matrix Metalloproteinase 2	3.582	1.92071E-14	Proteolysis, cell migration
Smad7	SMAD Family Member 7	3.533	1.20882E-14	Negative regulation of peptidyl-serine phosphorylation, negative regulation of TGF beta receptor signaling pathway
Gpx3	Glutathione Peroxidase 3	3.474	5.48921E-20	Oxidation-reduction process, glutathione metabolic process
Mmp9	Matrix Metalloproteinase 9	3.421	0.002724407	Apoptotic process, negative regulation of cation channel activity
Dusp1	Dual Specificity Phosphatase 1	3.173	3.2342E-261	Positive regulation of apoptotic process, negative regulation of DNA biosynthetic process
Fos	FBJ Murine Osteosarcoma Viral Oncogene Homolog	3.163	2.0595E-292	Cellular response to calcium ion, response to drug
Egr1	Early Growth Response 1	3.059	1.57477E-40	Positive regulation of chemokine biosynthetic process, positive regulation of interleukin-1 beta biosynthetic process
Fgf2	Fibroblast Growth Factor 2	3.052	0.001045603	Positive regulation of phospholipase C activity, positive regulation of blood vessel endothelial cell migration
Serpine2	Serpin Family E Member 2	2.239	4.85653E-06	Nervous system development, regulation of peptidase activity
Egfr	Epidermal Growth Factor Receptor	2.154	0.001338903	Positive regulation of fibroblast proliferation, regulation of peptidyl-tyrosine phosphorylation
Gas6	Growth Arrest-Specific 6	1.971	7.01844E-79	Positive regulation of ERK1 and ERK2 cascade, negative regulation of fibroblast apoptotic process
Nlrp3	NOD-like Receptor Family Pyrin Domain Containing 3	1.971	5.03474E-19	NLRP3 inflammasome complex assembly, positive regulation of interleukin-4 production
Ptger3	Prostaglandin E Receptor 3	1.962	0.020507062	Inflammatory response
Ptgs1	Prostaglandin-Endoperoxide Synthase 1	1.954	1.2782E-115	Response to oxidative stress
Sod1	Superoxide Dismutase 1	0.887	0.001433812	Negative regulation of inflammatory response
Ptger2	Prostaglandin E Receptor 2	0.887	0.066984764	Inflammatory response, cytosolic calcium ion concentration
Stat1	Stat1	0.885	8.76751E-05	cell migration, receptor signaling pathway via JAK-STAT
Sirt1	Sirtuin 1	0.859	0.021097814	Positive regulation of camp-dependent protein kinase activity
Sqstm1	Sequestosome 1	0.851	2.01199E-18	Interleukin-1-mediated signaling pathway
Casp1	Caspase 1	0.824	6.77483E-10	Cellular response to mechanical stimulus, positive regulation of I-kappa b kinase/NF-kappa b signaling
Gstp1	Glutathione S-Transferase Pi 1	0.823	0.044935984	Cell-matrix adhesion, acute inflammatory response
Gpx4	Glutathione Peroxidase 4	0.823	2.74223E-10	Regulation of inflammatory response
Timp2	Tissue Inhibitor of Metalloproteinases 2	0.800	5.24678E-23	Adenylate cyclase activity, positive regulation of MAPK cascade
Spp1	Secreted Phosphoprotein 1	0.758	2.8103E-132	Inflammatory response, cell adhesion
Gstm1	Glutathione S-Transferase Mu 1	0.744	6.30663E-38	Glutathione derivative biosynthetic process
Ccl4	Chemokine Ligand 4	0.716	1.57668E-17	Positive regulation of ERK1/2 cascade, calcium-mediated signaling
Vim	Vimentin	0.699	1.5086E-170	Intermediate filament organization, collagen biosynthetic process
Stat2	Stat 2	0.687	3.68207E-26	Receptor signaling pathway via JAK-STAT
Ccl3	Chemokine Ligand 3	0.682	1.51061E-44	Positive regulation of calcium ion import
Ccl2	Chemokine Ligand 2	0.615	1.0145E-142	Cellular response to fibroblast growth factor stimulus, cell adhesion
Tlr9	Toll-Like Receptor 9	0.584	6.49557E-06	Positive regulation of interleukin-18 production
Dusp10	Dual Specificity Phosphatase 10	0.562	0.000431134	Regulation of adaptive immune response, negative regulation of stress-activated MAPK cascade
Dusp8	Dual Specificity Phosphatase 8	0.539	0.018916559	Protein dephosphorylation
Alox15	Arachidonate 15-Lipoxygenase	0.445	0.022754094	Wound healing, ossification, positive regulation of heterotypic cell-cell adhesion, positive regulation of ERK1 and ERK2 cascade
Nfkbiz	Nuclear Factor Kappa-B Inhibitor Zeta	0.384	4.39923E-11	Regulation of transcription, transcription, DNA-templated
Ccl5	Chemokine Ligand 5	0.365	2.33658E-36	Calcium ion transport, cell-cell signaling
Irf7	Interferon Regulatory Factor 7	0.206	2.25856E-58	Immune system process, regulation of adaptive immune response, regulation of adaptive immune response

B

30 Mechanosensitive genes (WT vs. TRPV4 KO)				
Gene	Description	Fold Change	Pvalue	Related Biological function
Mmp8	Matrix Metalloproteinase 8	4.29	0.000001	Collagen catabolic process, proteolysis
Trpm2	Transient Receptor Potential Melastatin 2	3.70	0.0050859	Cellular response to temperature stimulus, ion transmembrane transport, calcium ion transport, positive regulation of insulin secretion, release of sequestered calcium ion into cytosol,
Mmp2	Matrix Metalloproteinase 2	3.58	1.921E-14	Negative regulation of vasoconstriction, bone trabecula formation, cellular response to amino acid stimulus, response to drug, regulation of cell adhesion
Trpc4	Transient Receptor Potential Canonical 4	3.53	1.179E-26	Regulation of cytosolic calcium ion concentration, calcium ion transport
Mmp9	Matrix Metalloproteinase 9	3.42	0.0027244	Positive regulation of angiogenesis, ossification, collagen catabolic process
Mmp15	Matrix Metalloproteinase 15	3.11	0.0002467	Proteolysis
Cald1	Caldesmon 1	2.89	1.477E-19	Regulation of smooth muscle contraction, actin filament bundle assembly, response to TGF beta, positive regulation of protein binding,
Trpm5	Transient Receptor Potential Melastatin 5	2.74	0.0122967	Regulation of ion transmembrane transport, cation transport
Mmp27	Matrix Metalloproteinase 27	2.52	1.4E-10	Collagen catabolic process
Mmp23	Matrix Metalloproteinase 23	2.16	0.0073346	Proteolysis
Itgal	Integrin Subunit Alpha L	2.02	1.46E-64	Cell adhesion, single organismal cell-cell adhesion, leukocyte cell-cell adhesion
Vcl	Vinculin	1.71	3.43E-54	Regulation of cell migration
Piezo1	Piezo Type Mechanosensitive Ion Channel Component 1	1.69	2.444E-97	Positive regulation of cell-cell adhesion mediated by integrin, cellular response to mechanical stimulus
Mmp13	Matrix Metalloproteinase 13	1.63	1.13E-294	Growth plate cartilage development, collagen catabolic process
Itgax	Integrin Subunit Alpha X	1.61	7.41E-235	Cytokine-mediated signaling pathway
Pxn	Paxillin	1.59	1.407E-24	Substrate adhesion-dependent cell spreading, cell-matrix adhesion
Trpm7	Transient Receptor Potential Melastatin 7	1.50	8.573E-32	Calcium-dependent cell-matrix adhesion, actomyosin structure organization
Actb	Actin Beta	1.45	9.2E-188	Cytoskeleton organization, VEGF receptor signaling pathway
Itga6	Integrin Alpha 6	1.42	0.0001122	Cell-matrix adhesion, positive regulation of cell-substrate adhesion
Itgb5	Integrin Beta 5	1.36	1.41E-17	Cell-matrix adhesion, integrin-mediated signaling, stress fiber assembly,
Itgav	Integrin Alpha V	1.27	2.3E-14	Extracellular matrix organization, positive regulation of MAPK cascade
Mmp14	Matrix Metalloproteinase 14	1.26	2.604E-08	Response to mechanical stimulus
Trpv2	Transient Receptor Potential Vanilloid 2	1.24	2.03E-18	Calcium ion transport, response to heat
Cd36	Cluster of Differentiation 36	0.96	0.0046804	Positive regulation of MAPK cascade
Mmp12	Matrix Metalloproteinase 12	0.93	4.229E-10	Tissue remodeling, wound repair, atherosclerosis, tumor invasion.
Tln1	Talin 1	0.93	0.0001706	Cortical actin cytoskeleton organization, integrin-mediated signaling pathway
Itgb2	Integrin Subunit Beta 2	0.84	1.677E-30	Toll-like receptor 4 signaling pathway, positive regulation of NF-kappa b transcription factor activity
Itgb3	Integrin Subunit Beta 3	0.69	1.177E-31	Wound healing, cell adhesion mediated by integrin
Itgam	Integrin Subunit Alpha M	0.64	1.438E-69	Signaling pathway, integrin-mediated signaling pathway
Mmp19	Matrix Metalloproteinase 19	0.53	0.0000001	Cell differentiation, collagen catabolic process

Supplemental Figure 4: Tables detailing fold changes, p-values, descriptions, and biological functions of differentially expressed fibrotic (A) and mechanosensitive (B) genes in WT and TRPV4 KO BMDMs grown inside 2% 3D microcapsules for 10-day in the presence of IL4 and GMCSF (25 ng/ml).
